# Migrants’ and refugees’ health: towards an agenda of solutions

**DOI:** 10.1186/s40985-018-0104-9

**Published:** 2018-09-24

**Authors:** Stephen A. Matlin, Anneliese Depoux, Stefanie Schütte, Antoine Flahault, Luciano Saso

**Affiliations:** 1 Institute of Global Health Innovation, Imperial College London, London, UK; 2 Centre Virchow-Villermé for Public Health Paris-Berlin, Paris Office, Paris, France; 3 Institute of Global Health, University of Geneva, Geneva, Switzerland; 4 University of Sapienza, Rome, Italy

## Abstract

Despite the greatly increased numbers of migrants and refugees worldwide in recent years, insufficient attention has been paid to addressing their health needs. While a variety of international instruments assert the right to health, in practice, migrants and refugees—especially those awaiting clarification of their status, such as asylum seekers and those without documentation—often fall in cracks between service providers and humanitarian relief programmes at national and regional levels.

This report provides a summary of the current state of knowledge regarding the health issues of migrants and refugees and of the extent to which they are being met. It highlights, through a series of case studies, the diverse approaches to policies, entitlements and services provided in different jurisdictions, ranging from regional (Europe) and country (Germany, Iran, Italy, Turkey, South Africa) levels to provinces and cities (Quebec/Montreal, Berlin). These provide evidence of successes and challenges and highlight areas requiring further effort, including in the domains of policy, service design and delivery, education and training, research and communication. They also underscore the challenges of highly neglected aspects such as mental health and the critical importance of developing cultural/transnational competence in the health professional individuals and institutions working with migrants and refugees.

Results from discussions taking place in an M8 Alliance Expert Group Meeting (Rome, 23–24 June 2017) and from the literature are synthesised to develop an ‘agenda of solutions’. This agenda aims to provide a comprehensive framework, which bridges humanitarian, ethical and rights-based imperatives to provide a framework for action to tackle this crucial area.

## Background

### A crisis of solidarity

The period 2014–2015 witnessed the largest and most rapid escalation ever in the number of people being forced from their homes. Millions of people fled conflict in Syria, Iraq, Afghanistan and Ukraine, as well as persecution in areas of Southeast Asia and sub-Saharan Africa, creating the highest level of displacement since World War II [1]. These were not isolated events, but part of a worldwide phenomenon that has seen massive displacements of people globally in the twenty-first century [2]. While countries neighbouring the sources of those displaced host the largest number of refugees, movements towards other destinations—especially high-income countries—also rose substantially. At mid-2016, Turkey hosted the largest number of refugees (2.8 million) of any country, while regionally, sub-Saharan Africa hosted 4.5 million and European countries (excluding Turkey) 2.1 million refugees in total [3]. Within Europe, Germany, Italy, France and Greece received the greatest numbers of asylum applications in 2016 [4, 5]. Humanitarian assistance rose to a global total of US$ 27.3 billion in 2016—the highest ever, but only 60% of the estimated amount needed [6].

With concern about the very large increases in numbers of migrants and refugees arriving at the borders of Europe [7] and elsewhere [8, 9] elevating the issue to the highest position on the political agenda of many countries (Table 1) [10], the United Nations (UN) Secretary-General, Ban Ki-moon, wrote [11] that ‘this is not a crisis of numbers; it is a crisis of solidarity. Almost 90 per cent of the world’s refugees are hosted in developing countries. Eight countries host more than half the world’s refugees. Just ten countries provide 75 per cent of the UN’s budget to ease and resolve their plight.’

**Table 1:** The refugee crisis in Europe, 2016

It is impossible to talk about health issues in the past year in Europe without reflecting on the refugee crisis, and the challenges and opportunities that it has presented for Europe. Over one million children, women and men arrived at our shores and borders last year. The European Union had a common responsibility to ensure that these persons, many of them physically and mentally exhausted, were offered care and support, including through the provision of healthcare when required. Jean-Claude Juncker, President of the European Commission [10]	

On 19 September 2016, for the first time, the UN General Assembly hosted a High-Level Summit to address large movements of refugees and migrants, with the aim of strengthening governance of international migration and creating a more responsible, predictable system for responding to large movements of refugees and migrants. The following day, US President Obama hosted a complementary Leaders’ Summit on Refugees, co-hosted by Canada, Ethiopia, Germany, Jordan, Mexico and Sweden, which appealed to governments to pledge significant new commitments on refugees [12].

The distinction between migrants and refugees is important in discussing these two sets of people.

- The International Organization for Migration (IOM) defines a migrant as any person who is moving or has moved across an international border or within a State away from his/her habitual place of residence, regardless of (a) the person’s legal status, (b) whether the movement is voluntary or involuntary, (c) what the causes for the movement are or (d) what the length of the stay is [13].
- According to the 1967 Protocol of the1951 Refugee Convention, a refugee is a person who, ‘owing to a well-founded fear of persecution for reasons of race, religion, nationality, membership of a particular social group or political opinions, is outside the country of his nationality and is unable or, owing to such fear, is unwilling to avail himself of the protection of that country.’ The 1984 Cartagena Declaration states that refugees also include persons who flee their country ‘because their lives, security or freedom have been threatened by generalised violence, foreign aggression, internal conflicts, massive violations of human rights or other circumstances which have seriously disturbed public order’ [14].
- Refugees are therefore a sub-set of migrants who are specially defined by their reasons for displacement and fear of consequences if they return and who are afforded special protection and entitlements by international agreements [15, 16]. A person who seeks safety from persecution or serious harm in a country other than his or her own and awaits a decision on the application for refugee status under relevant international and national instruments is classified as an asylum seeker. In case of a negative decision, the person must leave the country and may be expelled, as may any non-national in an irregular or unlawful situation, unless permission to stay is provided on humanitarian or other related grounds [17].

Beyond the technical definitions, the choice of term used by the media and politicians to describe those on the move and those arriving has become a topic of heated debate. Some commentators have suggested that by calling people fleeing war ‘migrants’, politicians hope to downplay their responsibility in caring for them. Deporting refugees is illegal under most circumstances and looks bad, while deporting unauthorised economic migrants can be represented as the state protecting its border and economy from those who have no right to be there under domestic law [18, 19].

At the September 2016 UN Summit, 193 Member States signed up to the New York Declaration [20]. Health is referred to at several points in the Declaration [paragraphs 30–33, 59, 80 and 83 and in paragraphs 5c, 7b and 13b of the annexed ‘Comprehensive Refugee Response Framework’]. These references encourage States to address the vulnerabilities to human immunodeficiency virus (HIV) and the specific health care needs experienced by migrant and mobile populations, as well as by refugees and crisis-affected populations, and to support their access to HIV prevention, treatment, care and support; commit States to combatting sexual and gender-based violence and providing access to sexual and reproductive health care services and to working to provide for basic health, education and psychosocial development; promise measures to improve integration and inclusion with particular reference to access to health care, among other services; reaffirm commitment to protect the human rights of migrant children, particularly unaccompanied ones, ensuring that the best interests of the child is a primary consideration in all relevant policies; commit to providing humanitarian assistance to refugees so as to ensure essential support in key life-saving sectors, such as health care, supporting host countries and communities in this regard; and commit to working to ensure that the basic health needs of refugee communities are met and that women and girls have access to essential health care services, to providing host countries with support in this regard and to developing national strategies for the protection of refugees within the framework of national social protection systems, as appropriate.

The onus of responsibility rests with States to respond to the health needs of migrants and refugees arriving in their own countries and to support those trying to meet the health needs of migrants and refugees in camps or transit locations on the way to their destinations. To date, it appears that solidarity from the international community is lagging far behind the commitments made in New York—for example, in the insufficient responses so far made to assist Uganda in managing the arrival of nearly one million refugees from South Sudan [21].

The challenge of migrants and refugees cannot be viewed as a short-term one that can be resolved exclusively by means of ‘exceptional’ or ‘emergency’ responses. The drivers that result in large-scale movements of people within and between countries are diverse, complex and interactive. Many of them are more likely to increase rather than decrease in the coming decades, including extreme weather events and slower shifts in weather patterns resulting from global warming that can lead to food and water shortages and losses of livelihoods and impacts of population increases, urbanisation, land degradation, deforestation and sea level rise. In addition, it can be expected that violence, political oppression and human rights abuses, as well as desires by people for a better life and greater economic opportunity, will continue to act as sources of involuntary or voluntary migration. It is therefore important to search for solutions that recognise migrants, refugees and asylum seekers as ‘part of society’ and that make them ‘structural’ rather than ‘external’ in health systems as well as other areas.

An extensive study on migration and health in Europe, published in 2011 [22], noted that ‘all too often, the specific health needs of migrants are poorly understood, communication between health care providers and migrant clients remains poor, and health systems are not prepared to respond adequately. The situation is compounded by the problems migrants face in realising their human rights; accessing health and other basic services; and being relegated to low paid and often dangerous jobs, with the most acute challenges being faced by undocumented migrants, trafficked persons and asylum-seekers. One major reason for this lack of understanding is the scarcity of data’.

Moreover, the study reported that there was a tendency in many European Union (EU) Member States to restrict entitlements of undocumented migrants to health services ‘to discourage the entry of new migrants’, with nine of 27 EU countries in 2010 restricting access to health services for undocumented migrants so that emergency care was inaccessible, only five offering them access to health services beyond emergency care and only four [Netherlands, France, Portugal and Spain] affording them entitlement to access the same range of services as nationals of that country [as long as they met certain pre-conditions, such as proof of identity or residence].

While examples of good practices in the treatment of health needs of migrants and refugees could be found, the study emphasised that, for long-term sustainability, structural changes were required that embed good practices in health policy and practice.

A series of reports from the Health Evidence Network of the World Health Organization (WHO) European Region (WHO-EURO) published in the period 2003–2016 summarises evidence available on diverse aspects of migrant and refugee health in Europe [23].

The large increase in displaced persons, migrants and refugees seen in 2014–2016 brought a new urgency to global efforts to achieve equity in access to health services. At the 69th World Health Assembly (WHA) in May 2016, Member States overwhelmingly supported the vision of a future where ‘all people have equal access to quality health services that are co-produced in a way that meets their life course needs, are coordinated across the continuum of care, and are comprehensive, safe, effective, timely, efficient and acceptable’ [24]. Implementation of this vision needs to address and include the health needs of migrants. WHO emphasises that the access of refugees and migrants to quality, essential health services is of paramount importance to rights-based health systems, to global health security and to public efforts aimed at reducing health inequities [25]. It notes, however, that access to health services is affected by poverty, stigma, discrimination, social exclusion, language and cultural differences, separation from family and socio-cultural norms, financial and administrative hurdles and lack of legal status [26].

WHO has observed that there is a need for reliable global data on migration and health, particularly in relation to undocumented migrants and those not accessing formal services [26]. In May 2017, resolution WHA 70.15 on ‘Promoting the health of refugees and migrants’ was endorsed at the 70th WHA [27]. The resolution urges Member States and requests WHO to identify and collect evidence-based information, best practices, experiences and lessons learned on addressing the health needs of refugees and migrants, in order to contribute to the development of a draft global action plan on promoting the health of refugees and migrants, to be considered for adoption at the 72nd WHA in 2019 and to report back to the WHA. WHO instituted an online survey, inviting Member States, institutions, networks, civil society groups, individuals and relevant organisations involved in refugees’ and migrants’ health, to provide relevant information, examples and lessons learned [28].

Reports from meetings organised by the Centre Virchow-Villermé Paris-Berlin [29, 30] and at the M8 Alliance’s World Health Summit Regional Meeting in North America [31] have also reviewed present knowledge and highlighted gaps in research.

Against this background, it is urgent to understand the nature of migrants’ and refugees’ health needs and the barriers that exist to meeting them, to learn from the successes and failures of different approaches and to develop new ones where required. The urgency is not only for knowledge and good practices in service provision, but also for policies that provide an effective framework for action in conformity with the UN Declaration, while engaging with and attracting broad support from the population. Running through all such practices and policies must be strong threads of humanitarian assistance and equity.

This paper draws on material presented (some of it hitherto unpublished) during the Expert Meeting on Migrants’ and Refugees’ Health organised by the M8 Alliance and held in Rome on 23–24 June 2017, supplemented by relevant literature.

### International frameworks on migration and health

Health implications of migration, whether forced or voluntary, have been a concern for centuries [32], with movements of people sometimes being restricted—for example, in medieval times due to fears of plague; in the nineteenth century when devastating cholera outbreaks swept from Asia into Europe and the United States of America (USA), leading to the International Sanitary Conferences [the forerunner of the modern International Health Regulations]; and in modern times, the imposition of official or unofficial quarantines in response to threats of pandemics [33].

Migrants’ health often remains marginal in broader discussions on migration, and migrants are a frequently forgotten population in health strategies. However, with increasing acceptance by states of their responsibility for ensuring health and human rights, fears about threats of infection are gradually giving way to a desire to treat both communicable diseases and the broader health problems of arriving migrants and refugees. Annex II of the New York Declaration set in motion a process of intergovernmental consultations and negotiations culminating in the planned adoption of a Global Compact on Migration at an intergovernmental conference on international migration in 2018 [34]. However, specific reference to health is missing among the areas for attention in the Global Compact that are highlighted [35] in UN General Assembly Resolution A/71/L.58. IOM argues [36] that health is a core cross-cutting theme in the follow-on to the New York Declaration and points out that there is a clear normative framework for the rights of migrants and refugees to health without discrimination, derived from, among others, the global human rights framework [37] and the WHO Constitution [38] and that a number of goals and targets of the 2030 Agenda for Sustainable Development [39] directly and indirectly promote migrant health.

WHO supports policies that provide health services to migrants and refugees, irrespective of their legal status [40], with the provision of adequate standards of care for refugees and migrants being important for population health and fundamental for protecting and promoting the human rights of both the refugees and migrants and the host communities.

While the UN Sustainable Development Goals principle of ‘leave no-one behind’ is inclusive of migrants and refugees, the realisation of universal health care for migrants and refugees requires evidence-based, inclusive policies that balance the costs and benefits of ‘health for all’ in a public health and development perspective [41]. At present, there is a lack of effective global governance for public health and a need for new governance structures that are beyond the present capacities of WHO and may have to evolve from elsewhere, such as the grass roots.

### The economic dimensions of migration

Historically, views about migrants have varied substantially over time, both between and within countries. A major factor in these variations has often been related to attitudes as to how migrants will impact on the economy of the host country. Since these attitudes influence the treatment of migrants and the extent to which they are accorded rights and/or assistance, including health services, they are significant for the present discussion.

Migration has a number of positive societal effects, including economic, employment and development benefits [42]. A publication by the World Bank [43] provided an overview of the economic benefits and challenges associated with migration, prepared as a contribution to the UN General Assembly’s Summit on refugees and migrants in 2016. It noted that migration brings large benefits to migrants and to the countries involved. Migrants from the poorest countries, on average, have experienced a 15-fold increase in income, a doubling of school enrolment rates and a 16-fold reduction in child mortality after moving to a high-income country. In the origin countries, migration lowers unemployment, opening access to more productive and higher-paying jobs. Migrants’ remittances offer tangible benefits to origin countries. In 2015, remittance flows to low- and middle-income countries reached US$ 432 billion, more than three times the size of official development assistance. Migration also facilitates trade, investment and transfers of technology. But migration may also involve costs, including the brain drain, associated with the migration, among others, of teachers, doctors and nurses. In the destination countries, immigration increases labour and skill supply, innovation and entrepreneurship. A report [44] from the Organisation for Economic Cooperation and Development (OECD) demonstrated that immigration provided a net positive fiscal effect. In the ageing societies, immigration of young workers could ease the strained pension systems and the burden of caring for the elderly. The World Bank report acknowledged that, despite the documented benefits of immigration, many people and policy makers in destination countries fear that immigration leads to loss of jobs, imposes heavy burdens on public services, erodes social cohesion and increases crime levels, whereas in practice, in many countries, migrants have net positive effect on government budgets and immigrants are less likely to commit serious crimes or be behind bars than the native-born. Another facet of the issue is that migrants have a more precarious position in the labour market and are more vulnerable to economic downturns than the general population [45].

## Main text

### Tracing pathways to migrants’ and refugees’ health

Migration often occurs over months or years with several steps along the way and a number of contributory factors involved. An example is the migration that began with the internal movement of people to Saint-Louis, northern Senegal, as a result of environmental shifts that degraded the land and destroyed their agricultural livelihoods [46]. Their arrival added to already unsustainable pressures in Saint-Louis, a coastal town heavily dependent on fishing but struggling due to coastal erosion, overfishing and a burgeoning population. Consequently, people in Saint-Louis have migrated [often irregularly] to Mauritania or have been enticed by smugglers to move on to other countries. Some have gone to Libya from where, pressured by very bad local conditions, they have risked their lives in unsafe boats and at the hands of unscrupulous smugglers to try to cross the Mediterranean to get to Europe. These people on the move could be categorised as environmental migrants, but in addition to land degradation, they have experienced political violence and economic and other pressures along the way.

The World Bank’s analysis recognises a number of drivers of migration, including income gaps and inequality, demographic imbalances and environmental change and suggests that migration pressures will continue for the foreseeable future. In 2015, the ratio between the average income of the high-income countries and that of the low-income countries stood at 70:1, a gap that it will take decades to close. Fragility, conflict and violence are also drivers of migration. Increased drought and desertification, rising sea levels, repeated crop failures and more intense and frequent storms are likely to increase internal migration and, to a lesser extent, international migration [43].

#### Framework for analysis: determinants of health

**Fig. 1:**
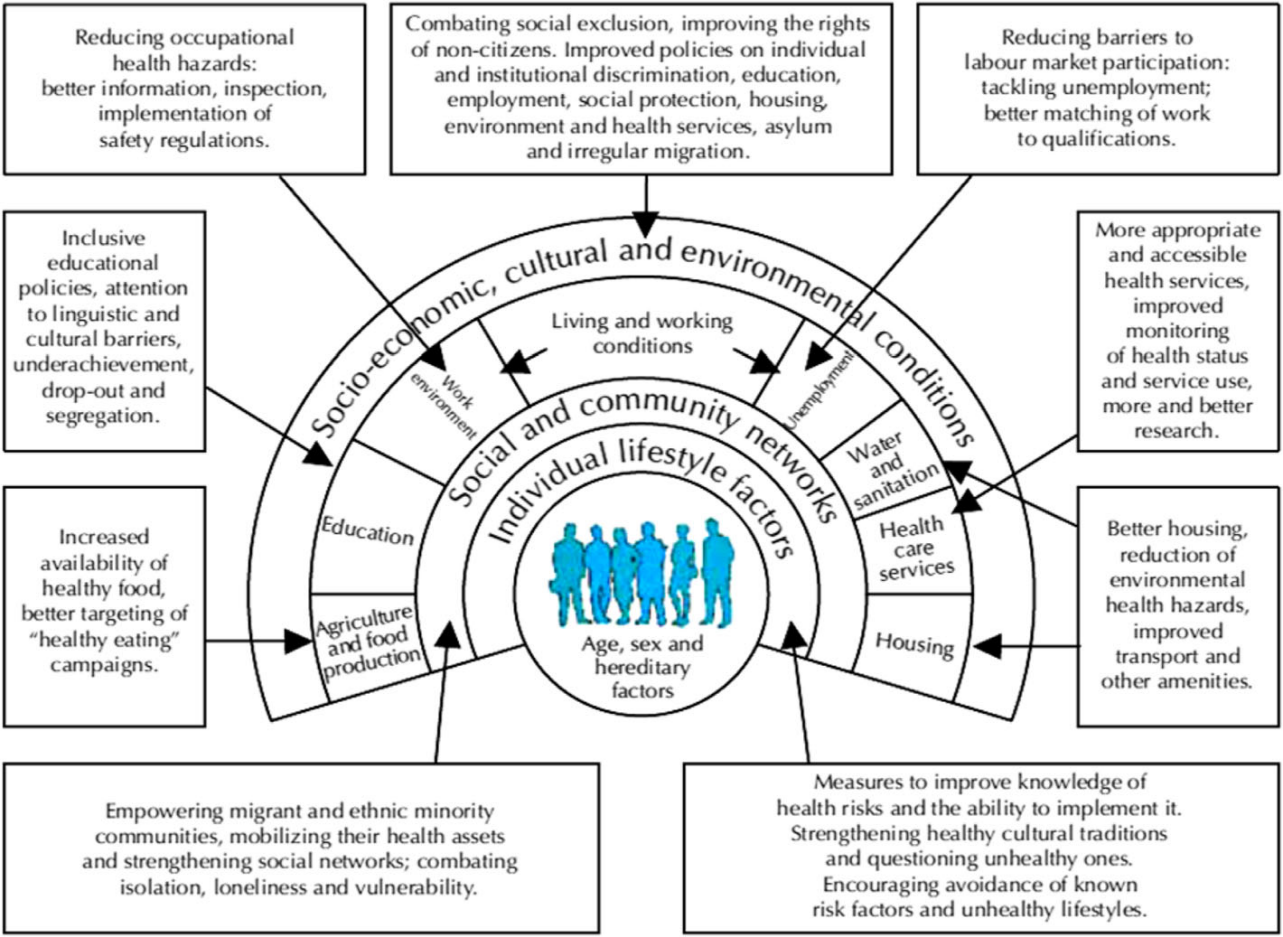
Policy measures required to tackle the social determinants of health for migrants and ethnic minorities. Source: [47]

It is evident that a comprehensive approach is needed, in which the health of the migrant is considered across disciplines, sectors and geographies and within contexts framed by political, social, economic and cultural factors as well as biological and medical ones. Thus, a useful starting point for policies on migrants’ health is the work that has been done on social determinants of health broadly, which can be adapted and focused to the specific circumstances of migrants and also applies to other marginalised groups such as ethnic minorities [47] as illustrated in Fig. 1 [48].

### Health before, during and after the migration process

**Fig. 2:**
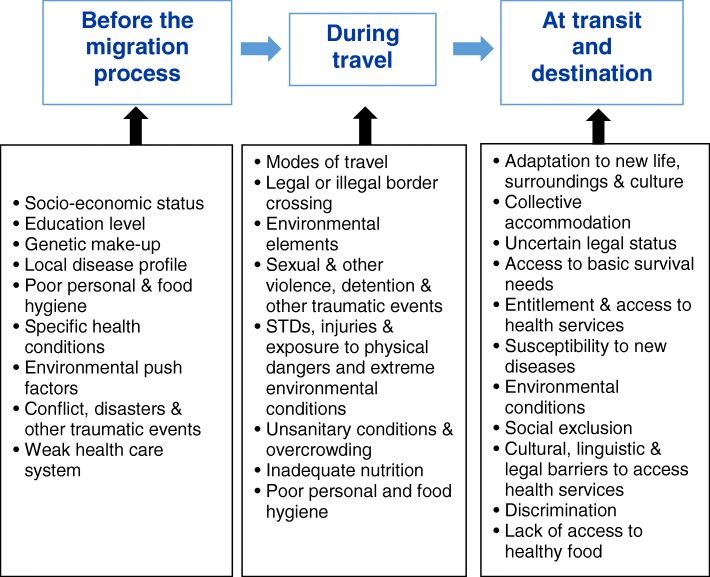
Risks to health of migrants

Risks to the health of migrants arise at every stage along their journeys, from before the migration process starts, during travel and at transit and destination points (Fig. 2). There are also health risks for those migrants who return home, including loss of ties and social networks, social attitudes to returnees and re-exposure to old risk factors. A dynamic analysis that considers temporal events and cumulative impacts of different determinants at different stages and phases is therefore necessary.

**Fig. 3:**
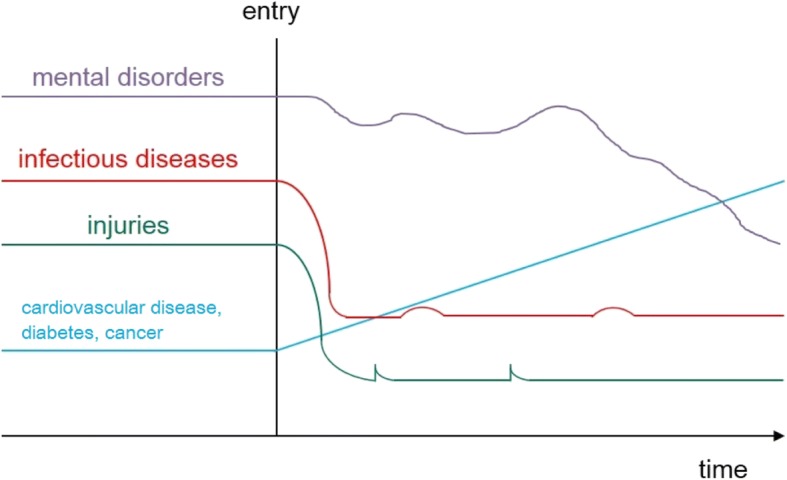
Time course: hypothetical scheme of different disease entities. Source: Alexander Krämer

The consequence of different temporally related events and of different treatments at various stages along the migrant’s pathway is that health issues will manifest at different stages and show different progression over time, as illustrated in the hypothetical scheme (Fig. 3). Infectious diseases and injuries acquired prior to or during travel are treated early on, but new diseases such as non-communicable and occupational diseases resulting from lifestyle shifts in the new location increase over time, and mental health problems emerge and resurface at intervals, as a result of traumatic events before or during migration and as longer-term stresses such as fear of deportation, separation from family, loneliness, isolation and social exclusion accumulate.

Prior to departure, the health status of migrants will usually reflect the characteristic health patterns of the countries where they resided. For low- and middle-income countries, these will typically involve relatively high levels of Group 1 diseases (communicable diseases and maternal, perinatal and nutritional conditions) compared with Group 2 diseases (non-communicable diseases (NCDs)) and Group 3 (injuries), the cause groups being those used in global burden of disease studies. However, local factors can greatly alter the disease distribution. For example, data from the Institute of Health Metrics shows that the burden of disease in Syria is highly unusual, with conflict-related factors moving from the 19th position to the 1st position between 2005 and 2015 as the main cause of death and disability [49]. Across the Middle East, deaths resulting from violence grew by 850% between 1990 and 2015 and the incidence of many chronic diseases also rose dramatically; the death rate from diabetes, for instance, grew 216% over the period, according to a series of reports published in August 2017 [50].

In terms of impact of incoming migrants on the health profile of the receiving countries, it is often observed that immigrants arriving in the host country are healthier than comparable native populations but that the health status of immigrants may deteriorate with additional years in the country. The ‘healthy migrant effect’ is explained through the positive self-selection of immigrants and the positive selection, screening and discrimination applied by the host countries. The effect may be absent in refugees whose pathways to a destination country have included prolonged residence in refugee camps or arduous journeys. In the longer term, the health of migrants reflects changes in lifestyle, diet and environment in the host country, for example leading to increases in cardiovascular disorders [51, 52].

**Fig. 4:**
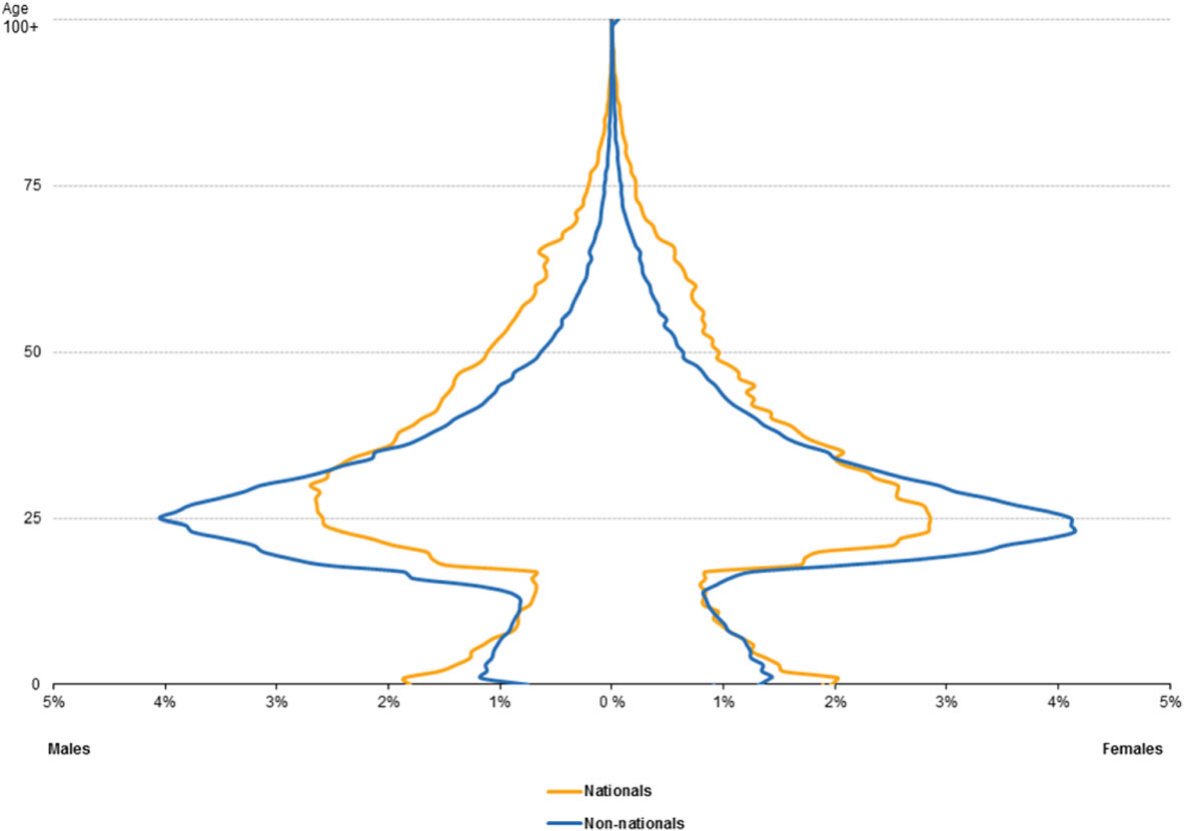
Age structure of immigrants by citizenship, EU-28, 2015. Source: Eurostat online data code: migr_imm2ctz; [53]

In addition to the epidemiological transition, migrants can have a significant effect on the demographic profile of the receiving country. Many high-income countries, e.g. in Europe, have been experiencing a major demographic transition characterised by smaller families and ageing populations, while low- and middle- income countries, e.g. in Africa, have seen relatively high (but declining) fertility rates and improving survival rates, resulting in expanding populations of young people. The overall demographic profile of non-nationals in Europe is therefore significantly younger than that of the national population (Fig. 4) [53]. Overall, there are transitions in risk factors affecting both migrant and receiving populations, impacting on health and health services.

The approach to addressing the health needs of migrants and refugees therefore needs to work along two tracks:

i. Coping with the incoming people and adapting in periods when there are exceptionally high numbers arriving at borders. This should include prophylaxis, screening and triage at the borders and in reception centres, and involve health assessment [54] and vaccines for people coming from countries affected by endemic infections and/or because of exposure to infectious agents in different contexts during their journey.
ii. Providing longer term equitable access to health promotion, disease prevention and care, including health care in camps and transit or detention centres for migrants as well as provisions for access to health services in the community.

Two levels of action are needed to support this approach, one requiring political and strategic measures to define a clear and transparent framework for countries, institutions and other actors and the other involving on-site measures (e.g. adequate shelter, sanitation and water supplies, safe food and adequate nutrition, providing access to vaccinations and medical care) to ensure that migrants’ health needs are addressed.

Ensuring equitable access to health promotion, disease prevention and care requires identifying and removing barriers, which may be associated with legal status and entitlement, or economic, social or cultural factors. These may be compounded by additional issues such as poor disease surveillance or political opportunism. The impact of the operation of these barriers may not only be seen in the equity and quality of health care received by the migrants and refugees, but also in the avoidable use of emergency care, which has economic consequences.

#### Addressing the causes

Root causes of the displacement of people that result in outward migration and create refugees and asylum seekers include some that can be addressed through international action—e.g. relating to conflicts, human rights abuses, accelerated global warming, massive inequities in economic opportunities and social freedoms—but only with difficulty and requiring long-term effort and commitment.

Ultimately, it is the solution to these challenges that will diminish the drivers for people to move over long distances. The EU has recognised this in the Communication on ‘A European Agenda on Migration’ from the European Commission (EC), which emphasises the importance of trying to halt the human misery created by those who exploit migrants and using the EU’s global role and wide range of tools to address the root causes of migration [55]. The World Economic Forum has pointed to the need for greater efforts to provide people at risk with protection and assistance in their own countries, reducing the need to flee in the first place and has highlighted the importance of implementing frameworks regarding the ‘responsibility to protect’ [56] that have been developed for protecting internally displaced persons [57].

As noted in the WHO-EURO high-level meeting on refugee and migrant health, many of the health, social and economic challenges associated with migration are the product of global inequity. Action that focuses solely on host countries will be less effective than integrated global, interregional and cross-border interventions and programmes, including in public health [58].

### The nexus between climate change, migration and health

With traditional landscapes and livelihoods of entire communities being increasingly threatened by climate change and extreme weather events, there is a pressing need to study the impact on human migration and population displacement. The first illustrated publication mapping this complex phenomenon describes the multiple factors at play and the challenges and highlights the opportunities [59].

**Fig. 5:**
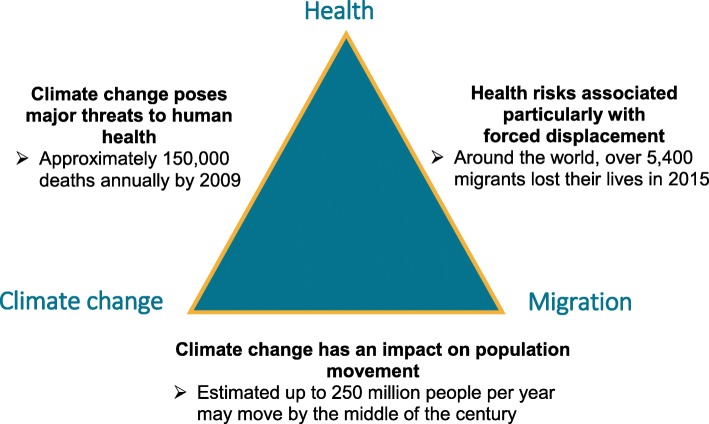
Links between climate change, health and migration

Figure 5 summarises in broad terms the relationships that are considered to operate: climate change has direct and indirect effects on population movement [12, 60], and also on health [61–63], while migration is itself a risk factor [23] for adverse health events. As pointed out by Beck, the multiple, diverse factors are all connected together in complex ways [64].

#### Scoping the scientific literature

As a starting point for identifying research gaps and opportunities for strengthening linkages between research and education, policy and practice, a scoping study of published literature was undertaken, as part of Centre Virchow-Villermé’s research project ‘4CHealth’ [65]. Preliminary results of a bibliometric analysis of the PubMed and Web of Science databases showed that, since the first scientific article mentioning migration and health appeared in 1945, the number published annually grew to more than 40,000 in 2016, with most of the increase occurring from 2000, and since the first article mentioning climate change and health appeared in 1956, the number published annually remained very low until around 2005 and then rose appreciably to nearly 5000 in 2016. However, only about 250 articles mentioned all three areas of climate change, migration and health.

Preliminary conclusions from the study are that existing research in the field is very weak. Potential health effects discussed in papers are frequently extrapolated from similar migration situations, while few real data exist. Regarding climate change, research so far has focused mainly on trying to estimate the numbers of people that will be displaced. For example, according to one study, future weather anomalies are expected to lead to an additional annual displacement of 11.8 million people in sub-Saharan Africa alone by the end of the twenty-first century [66]. Little research has been done on the health impacts of climate change and the potential multiplying effects of migration and climate change on health.

#### Scoping the media: migration as an adaptation strategy

The press has played an important role in the evolution of attitudes towards migrants and refugees, including those displaced by climate change. It has contributed to informing and shaping opinions, public responses and government policies, as well as reflecting popular sentiment and political stances.

A study of press coverage of the refugee and migrant crisis in the EU analysed press content in five European countries (Germany, Italy Spain, Sweden and United Kingdom (UK)) in 2014 and 2015 [67]. It observed that health was generally treated in the context of the burden on health systems and threats from infectious diseases posed by migrants and refugees. In particular, one notable finding was the high incidence of discussion of threats to welfare/health systems in the UK press (18.3%) which was much greater than the other countries in the sample (Sweden 11.4%, 7.9% Germany, 7.3% Italy, 6.7% Spain). Issues of post-arrival integration were covered in any depth by only a few newspapers. One Swedish paper, in April 2015, discussed in detail how to enhance refugees’ possibilities for employment once in Sweden and highlighted the advantages of offering language courses and fast-tracking foreign-born doctors trying to obtain their medical licence to practice in the country.

Often, when impacts of climate change and other events on displacement and migration are discussed, migrants are portrayed as expiatory, powerless victims. In health terms, migration is often seen as associated with health crisis [68], disease and death, as illustrated in the IOM’s Missing Migrants Project [69] and consequently framed as a public health emergency [70].

In recent years, climate change has been increasingly mooted as a possible adaptation strategy. This approach has been strongly advocated by different international organisations, keen to promote a more positive view of migration. According to Kanayo Nwanze, President of the International Fund for Agricultural Development [71], ‘The media, whether local or global, are among the world’s most influential institutions and how they shape the climate change narrative remains vitally important. If the world becomes aware of how climate change threatens our food security or why it is a catalyst for migration and conflict, then we can expect better support for policies and investments that can pre-empt future crises.’ Yet in public debates, migration associated with climate change remains overwhelmingly presented as a disaster in the making, a humanitarian catastrophe to avoid at all costs.

Little work has been conducted hitherto to try to examine and understand the role of the press in relation to climate change, migration and health. Previous studies have suggested that a lack of basic knowledge on climate change is one of the largest perceived barriers to taking action [72] and that the media’s framing of the issue has a critical influence on the perception of urgency and willingness to respond [73]. These and other studies provide insight into the communication methods that are most effective in inducing behaviour change [74–77] and, in particular, indicate that framing climate change as a public health concern rather than as an environmental issue is one of the elements that would help increase engagement of the public with climate change.

In a study undertaken by the Centre Virchow-Villermé, treatment of the theme of migration as an adaptation strategy is being examined through a comparison of the leading French and German newspapers, *Le Monde* and *Frankfurter Allgemeine Zeitung* [78]. The analysis examined how these newspapers framed the relation of climate change and health between 1990 and 2016 and what kinds of recommendations and solutions were stated to address the health impacts of climate change. The study undertook bibliometric analysis of publications in the databases of the two newspapers, by tracking the use of key climate terms and their co-occurrence with ‘migrants’ and ‘refugees’. Within the data set, it was observed that the terms refugee, migrant and displaced were used in equal proportions.

Among other aspects, the analysis examined the range of voices in the articles. Few health professionals were quoted, the largest groups of voices being those of politicians, researchers and climate professionals and experts. Absent were migrants, who appear to have no direct voice in the debate about their status and treatment.

The frequency of articles related to climate change and migration showed a big spike in 2015 in both newspapers, related to the 2015 UN Climate Change Conference ‘COP 21’. In *Le Monde*, the time course of 56 articles [1996–2015] showed four distinct phases, with the first articles (1996–2007) in the period studied flagging the subject within other contexts, while from 2007, the subject began to be treated on its own, from 2009, it became complex and transdisciplinary and in 2011–2015, migration was presented as an adaptation strategy to cope with climate change impacts. Fewer (26) articles appeared in the *Frankfurter Allgemeine Zeitung*, with a cluster (11 articles) peaking in 2015. Starting in 2012, migration was presented as a potential adaptation solution to climate change impacts. Articles in the German paper highlighted that ‘climate refugees’ are not accepted by international law; that it is difficult to argue that migration is caused by environmental change, as cause and effect are often not directly connected; that refugees resulting from climate effects are often seen as economic refugees; and that climate is only one of a number of triggers for migration (but as important as political causes). It was argued that the UN High Commissioner for Refugees (UNHCR) needs to update the definition of refugees.

It was noted that articles on migration as an adaptation strategy were not given prominent placement such as on front pages.

Overall, the analysis concluded that migration as an adaptation strategy has emerged as a major way of presenting the migration and climate change issue in the two newspapers but lacks a clear connection with health issues, so that these three topics are not framed together and that health implications of migration, such as the access to health care, are totally neglected.

### Policies in a regional and global context

#### Framing

According to international agreements, the responsibility for addressing the needs of migrants and refugees rests in the first instance with the countries in which they are located temporarily or long-term. More broadly, however, there are reasons for the responsibility to be shared by the international community, both on humanitarian grounds and as a matter of self-interest to enable migrants and refugees who have been displaced from their homes to find acceptable living conditions, livelihoods and opportunities as close as possible to their point of origin and to integrate peacefully and productively wherever they settle. Policies are therefore essential at national, regional and global levels that ensure the rights of migrants and refugees and that assist them with essential factors such as food, shelter health, services and opportunities for livelihoods.

In practice, health policy-making in the context of migration has often been viewed either in terms of its ‘threats’ to public health or from a rights-based approach that focuses on health hazards faced by individual migrants and the associated service challenges [79]. The rights-based perspective is more recent and grounded in medical ethics. It recognises migrants’ special vulnerability to, for example, interpersonal and occupational hazards, social exclusion and discrimination, as well as emphasises the importance of universal access and culturally competent health care services.

Such policies need to originate in a framing of migrants and refugees as people displaced by diverse circumstances outside their control, using migration as an adaptation strategy for survival. In practice, migration as an adaptation strategy has been discussed principally in relation to the impact of climate change. Migration is recognised as an environmental policy in the UN’s 2010 Cancun Adaptation Framework, in which paragraph (f) calls on Parties to undertake ‘measures to enhance understanding, coordination and cooperation related to national, regional and international climate change-induced displacement, migration and planned relocation, where appropriate’ [80]. Similarly, the Nansen Initiative, an intergovernmental process which resulted from a UNHCR Ministerial Conference in December 2011 at which Norway and Switzerland pledged to address the need for a more coherent approach to the protection of people displaced across borders in the context of disasters and the effects of climate change, has sought to recognise migration as a potential policy solution [81].

Gemenne [82] has described this as a paradigm shift: that migration in the context of climate change was no longer a disaster to avoid at all costs but a strategy that ought to be encouraged and facilitated. The movement of people was no longer a matter of migration policy but rather of environmental policy—an adaptation strategy.

The acceptance of this policy orientation at the international level stands in contradistinction to what, in reality, has been the evident policy of many countries, who have framed [or accepted a framing presented by media and populism] migrants and refugees as a threat, as ‘others’ who should be excluded and not given entitlements to national resources or assistance. There has been a prevailing ‘paradigm of immobility’ [83], regarding migration as an abnormality.

**Fig. 6:**
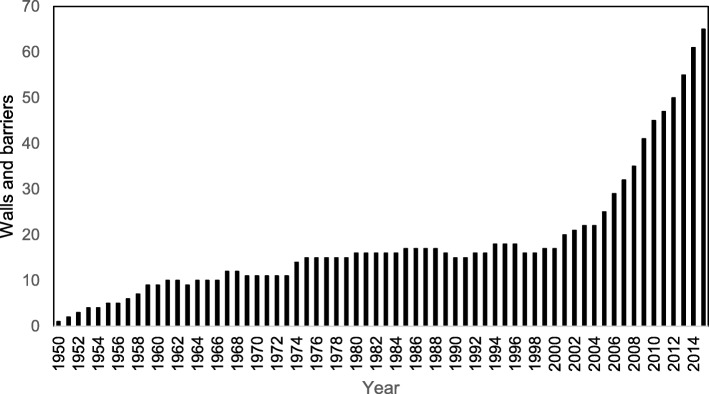
Number of walls and barriers standing or being built, 1950 to 2015. Redrawn from data extracted from [85–87]

In the extreme, this has led to a policy response of building walls and fences in a ‘keep them out’ syndrome [55, 84], with a large increase occurring since 2005 (Fig. 6) [85–87], as a physical manifestation of exclusion policies to protect the security and integrity of the state. Barriers make migration more dangerous and present a health challenge, since people will continue to migrate under pressure [e.g. due to perceived or real inequalities or threats to safety].

In practice, moving from framing migration as an adaptation to constructing policies to take this approach forward is challenging. Migration is not cost-free for poor people in constrained situations and also has impacts on the families and communities they leave behind, while it has political, social and cultural implications for the countries that will receive them as well.

Policies must examine the implications for different sectors, with implications for planning, budgeting and implementation. They must also reflect the reality that there is no such thing as a homogeneous society and that diversity exists without migrants, so that inclusive health systems must be the bedrock of health care provision. Consequently, it is important that policies should recognise migrants, refugees and asylum seekers as ‘part of society’ rather than requiring ‘exceptional’, ‘emergency’ responses, embedding them structurally in health systems and other areas at local, national and regional levels and addressing their needs as individuals and not as part of a plural ‘them’.

As noted by Diaz et al. [88], emphasising the health of migrants is vital for primary care because (1) caring for migrants and refugees serves to fulfil human rights and to orientate the ethical compass, through prioritising care for the most needy; (2) caring for migrants is vital in upholding social cohesion, particularly in societies with a high number of immigrants and refugees; and (3) providing good health care for migrants is an economic investment over the longer term.

#### Coherence

While migration has been expanding in recent years, there has not been commensurate development of policy approaches that are coordinated to address the associated health implications [89] Internationally, policy-making on migration has generally been conducted from policy sector ‘silos’ [e.g. international aid, security, immigration enforcement, trade and labour] that rarely include the health sector and which often have different, if not incompatible, goals [90].

Within the health arena, this lack of coherence is manifested in diversity in areas such as entitlements, services provided and disease surveillance, both within countries (e.g. Germany, Canada) and between countries (e.g. in the EU). There has also been a considerable lack of evenness in the degree of attention given to different health problems within each jurisdiction, with mental health often being highly neglected compared with other health issues.

Poor policy coordination and contradictory policy goals, such as increasing foreign labour requirements while maintaining restrictive rights for migrants, can exacerbate risk conditions related to migration and pose health challenges [91].

#### Ethics and the health of migrants and refugees

In general, the operation of a health ethics framework needs to relate to, among others, principles of ‘do no harm’, equity and human rights including the right to health, as well as special protections and rights accorded to females, children and persons with disabilities [92, 93]. Application of these principles to addressing the health of migrants and refugees (Table 2) [94] raises a number of major challenges, including policy questions of who is responsible and how much should be provided to those without means and practical considerations of how to ensure access to and uptake of entitlements and equity of treatment at the point of care. The medical and public health community has a responsibility to defend the principles and not to be silent about or complicit in abuses or omissions in policy or practice [95].

**Table 2:** Ethics and the health of migrants and refugees

The issues surrounding health and migration are important for a number of key reasons. They not only relate to the ethical implications of unequal access to health care but also are linked to (avoidable) costs to health systems and wider society. As a result, there is not only an ethical imperative to address issues of health and migration but also direct and indirect incentives, such as improved health, social cohesion, economic sustainability and political cooperation. Zsuzsanna Jakab, WHO Regional Director for Europe: Ref. [94]	

An overview [96] of approaches to the health of migrants from a public health ethics perspective noted that ‘there is little consensus between or within countries about an acceptable standard of health care for different migrant groups, such as undocumented migrants, asylum seekers, refugees and temporary workers. There is also considerable disagreement about how this health care might be accessed, or the philosophical and human rights positions that underpin discussions concerning access and delivery.’

An examination [97] of ethical issues in immigrant health care and clinical research highlighted four important areas of ethical concern for refugee and immigrant health care: (1) the relevance of cultural beliefs and values for ethical problems that arise in patient care, in relation to respect for persons and the articulation of individual autonomy; (2) problems associated with communication, with special attention to ethical issues surrounding the use of interpreters; (3) ethical dimensions of access to care, especially problems associated with discrimination and political repression; and (4) ethical problems associated with clinical research, with a particular concern about the application of informed consent and the protection of confidentiality. Practical guidelines were offered for clinicians facing ethical dilemmas in cross-cultural interactions with patients. It was argued that three elements are essential in successfully resolving moral problems in cross-cultural patient care and clinical research: an ability to communicate effectively with patients and their families, sufficient understanding of the patient’s cultural background and identification of culturally relevant value conflicts.

A systematic review [98] of available academic evidence and grey literature addressed the question of what policies and interventions work to improve health care access and delivery for asylum seekers and refugees in the European Region. It observed that access to health care is shaped by legal frameworks governing the rights of refugees and asylum seekers and by the regulation of the migration process. Other barriers in accessing health services include communication difficulties (e.g. lack of interpreters), cultural issues (e.g. gender preference for doctors), structural problems (e.g. transport) and bureaucratic barriers (e.g. social insurance systems). Access to specialist services can also be difficult. The nature and length of the asylum process plus the use of detention and dispersal can have a significant impact upon health outcomes. A good resettlement environment, including employment, family reunion, protection from discrimination and support for integration or repatriation, is associated with better health outcomes. Policy considerations include that improved information and documentation is needed to support the design of national and international minimum standards and management strategies in the health and social care of refugees and asylum seekers. Policy options include improved access to services by removal of legal restrictions; provision of full health coverage for all pregnant women and for children regardless of immigration status; adoption of approaches to improve communications, such as provision of interpreters and good documentation for patients; and adjustment of health care provision to improve service utilisation, for example allowing longer appointment times and the provision of transport.

Special protections and rights are afforded to refugees and to minors and their families. The medical profession is often asked to undertake examinations of claimants, which must be with their consent, to help identify or confirm signs indicating past persecution or serious harm, or to establish probable age.

Medical examinations should be performed by qualified medical professionals, acting objectively, impartially and gender-sensitively and be part of legally safe and efficient asylum procedures, assuring that similar cases are treated alike and result in the same outcome. They should be adequate and complete, allowing the identification and documentation of symptoms and signs of torture or other serious acts of physical or psychological violence, including acts of sexual violence, and based on the UN Manual on Effective Investigation and Documentation of Torture and Other Cruel, Inhuman or Degrading Treatment or Punishment (Istanbul Protocol) [99].

Article 18 of the 2013 Directive [100] of the European Parliament and Council states that, where deemed relevant by the appropriate authority, Member States shall, subject to the asylum applicant’s consent, arrange for, or allow the applicant to arrange for, a medical examination concerning signs that might indicate past persecution or serious harm. Article 25 of the 2013 Directive states that Member States may use medical examinations to determine the age of unaccompanied minors applying for international protection where there are doubts concerning the applicant’s age. Article 25 also states that the decision to reject an application for international protection by an unaccompanied minor who refused to undergo a medical examination shall not be based solely on that refusal and requires that ‘the best interests of the child shall be a primary consideration for Member States when implementing this Directive.’

One area of ethical concern has been the use of medical X-rays for the determination of age. Some individuals and groups (e.g. including a Member of Parliament in the UK) [101] have called for it to be used where there are doubts about the age of an applicant claiming to be a minor. However, there is clear evidence and a strong scientific consensus that this is an inaccurate method for assessing age and the UK Home Office have accepted this position [102]. The UK’s British Dental Association has strongly opposed the use of X-rays for age determination [103, 104], not only on scientific grounds but also because they believe it to be inappropriate and unethical to take radiographs of people when there is no health benefit for them, since it exposes them to risk of harm. Moreover, they assert that X-rays taken for a clinically justified reason must not be used for another purpose without the patient’s informed consent, without coercion and in full knowledge of how the radiograph will be used and by whom.

International legislation also recognises ‘vulnerable persons’ among asylum seekers, who are often exposed to a heightened risk of harm and thus require special care, support and protection. An individual’s classification as a ‘vulnerable asylum seeker’ can have important implications in the reception procedures, in the decision-making phase and in the definition of therapeutic needs and rehabilitation. Ethical methodology for the identification of vulnerable asylum seekers has been proposed [105].

#### Integration or separate services?

There are divided views and practices regarding the issue of whether integration or separation is better in the provision of migrants and refugees with health services [as well as in other key domains such as education, housing and the labour market]. The IOM’s 2010 World Migration Report [106] identifies integration as one of the six major challenges facing national governments in managing migration. Among the challenges in this area are definitional issues of ‘integration’ and ‘citizenship’, the impact of racism and xenophobia, the historical evolution of social and cultural factors and experiences of migration and of ‘health tourism’ in different places, the nature of the social welfare and health service delivery models in operation, and divergent attitudes among migrants and refugees themselves, the combination of all of which can result in a strong politicisation of the question [107].

A further challenge relates to the heterogeneity of the populations concerned. In Europe, for example, many migrants have been attracted to work in the health services—but because of their temporary and sometimes precarious status, they [especially women in lower-paid occupations] often experience a higher workload, working more at nights and in less secure employment. Access to health care for migrants as residents is sometimes limited because of confusion about the system and the failure of health care providers to be effective in explaining how health systems are structured and the extent of people’s entitlements. Migrants often feel that providers and practitioners show a lack of sensitivity to cultural aspects of health care, including views about conditions, ways of accessing health care and negotiating care arrangements. For newly arrived migrants [often undocumented] and refugees, questions of entitlement and provision depend heavily on local factors, and the utility and quality of services can be critically influenced by the availability of interpreters as well as health staff and facilities.

Important observations on this complex issue include the lack of research and data [especially evidence regarding what works best in local contexts], inconsistencies in policies and practices that are compounded by inadequate training of those involved in service design and provision, the need for provision of much more information to migrants on the availability of health care and their rights and entitlements and the absence of the voices of migrants and refugees in the debate and in the design and delivery of services to meet their needs.

A systems perspective on this issue is necessary, which develops and adapts approaches that are relevant at different stages along the pathway of arrival and settlement of migrants and refugees, with vertical services and/or mainstreaming being used flexibly and account taken of general systems capacities in relation to special needs.

### Mental health: treatment and rehabilitation strategies

Mental health problems may arise from events and conditions experienced before, during and after migration, including experiences of conflict, injury, violence, witnessing others including friends and family members being abused or dying, perilous journeys, hunger, confinement, harsh treatment by authorities, boredom, worries about health and about family back home and fears of being sent home [108]. Traumatisation may be severe but not always manifest at the point of arrival, as these can be ‘invisible wounds’ [109, 110].

Post-migration living difficulties can significantly increase the risk of post-traumatic stress disorder in immigrants [111, 112]. A study in Italy in 2009, carried out at the primary care outpatient service of the Caritas Health Service, a charity organisation providing free medical assistance to immigrants and people in social difficulty, examined first-generation immigrants. Potentially traumatic events, post-migration living difficulties, post-traumatic stress disorder and related conditions (anxiety, depression and somatisation) were frequent among immigrants in primary care, and either potentially traumatic events or post-migration living difficulties significantly influence resulting psychopathology. Implications for clinical practice included that general practitioners (particularly those working in services for immigrants) should pay particular attention to mental health screening in first-generation immigrants, whether or not they had the status of refugee. Early detection may offer the opportunity to reduce psychopathological morbidity, to avoid the risk of iatrogenic effects in non-recognised post-traumatic stress disorder and to implement appropriate treatment approaches [113]. Somatisation (medically unexplained symptoms) was found to be significantly related to traumatic events and post-traumatic symptoms among immigrants receiving primary care services [114].

The WHO ‘mhGAP Intervention Guide’ provides a technical tool for health care providers, decision-makers and programme managers involved in meeting the needs of people with mental, neurological and substance use disorders, in non-specialised health settings [115]. However, it does not specifically address migrants and refugees.

A study in 2014–2015 of asylum seekers landing in Sicily found that their medical conditions had been assessed on arrival but their mental health needs had not been addressed in any way, despite the likelihood of serious trauma before and during migration. Médecins sans Frontières provided mental health assessment and care for recently landed asylum seekers in Sicily. Half of the 385 individuals who presented themselves for mental health screening during the study period were identified and diagnosed with mental health conditions. Most were young West African males who had left their home countries more than a year prior to arrival. The most common conditions were post-traumatic stress disorder (31%) and depression (20%). It was concluded that mental health and psychosocial support and improved living circumstances should be integrated into medical and social services provided in the receiving countries [116].

Cultural differences can be a significant factor leading to misdiagnosis, for example in the recognition of schizophrenia and psychoses in post-traumatic stress disorder, as against immigrants’ ‘spiritual’ experiences of suffering and interpretation as possession.

There is an ongoing need for development of culturally appropriate mental health services for socially under-included and marginalised populations [117]. One much neglected area has been attention to children traumatised by conditions of conflict and disasters, as highlighted by the Children and War Foundation [118].

Overall, treatment strategies to address mental health issues in migrants and refugees need to recognise invisible wounds, provide supportive social facilities, expedite the evaluation of the asylum request (including legal support), provide for flexibility in treatment (e.g. in the setting and number of persons included in the therapy) and involve a multi-professional team including cultural mediators and anthropologists, with multi-level competencies (cultural issues, techniques to treat post-traumatic symptoms, psychopharmacology, etc.).

Conclusions are that most mental suffering in migrants is an understandable reaction to difficult living conditions, which appreciably influence onset, course and outcome. There is a significant number of immigrants (and refugees in particular) experiencing post-traumatic symptoms and a problem of differential diagnosis (post-traumatic and cultural syndromes mimicking schizophrenia) with a consequent risk of over-diagnosis of psychoses. Treatment should be organised by a multi-disciplinary team with expertise in several issues (cultural idioms of suffering, post-traumatic reactions, etc.). Improvement of resettlement conditions is a critical factor in achieving efficacious treatment.

Provision for mental health issues in migrants and refugees is especially challenging in the context that, in most if not all countries, the general level of provision for mental health issues is relatively weak and the resident population may have poor access to, or long waiting times for, services for diagnosis and treatment.

#### Supporting wellbeing with the arts

Given the levels of trauma, living difficulties, anxiety and depression experienced by many migrants and refugees, innovative approaches to assisting them to engage in activities that have positive psychological impact can be extremely valuable, including the use of participatory arts [119].

There is a growing body of research on the arts and wellbeing. Creative activities can benefit health and wellbeing through positive social experiences, leading to reduced social isolation; opportunities for learning and acquiring news skills; calming experiences leading to decreased anxiety; increased positive emotions such as optimism, hope and enjoyment; increased self-esteem and sense of identity; and increased inspiration and opportunities for meaning making [120–123]. Automatic movement such as knitting may facilitate access to the subconscious and could aid treatments such as cognitive behavioural therapy, for example. Performing a repetitive visual spatial task during or shortly after a traumatic event significantly cuts down the incidence of flashbacks [124].

Research being undertaken at a refugee camp in Jordan has aimed to develop a method for assessing the psychosocial impact of creative and cultural activities with displaced people [125]. Preliminary findings are that participation can be beneficial to people, who gain technical, life and social skills; there is a positive social aspect, generating friendship, sense of community and belonging as an antidote to loneliness and safe space, and a personal aspect, with improved mood, block of negative thoughts, an increased sense of ‘normality’, development of different identifications (beyond ‘victim’ or ‘asylum seeker’) and improved self-expression.

### Roles of non-governmental actors and education as a tool

#### Non-governmental actors

Non-governmental actors play extremely important roles in addressing the health challenges of migrants and refugees. They not only compensate for deficiencies in the services and resources provided by governments (e.g. through civil society voluntary groups, charities and non-governmental organisations (NGOs)), but also fulfil a host of functions that are complementary to the capacities of governments. These include providing research, analysis and independent policy advice (e.g. through academia, think tanks, foundations) and having the capacities to take longer-term views, to work innovatively and flexibly, to operate non-politically in contested spaces and to gain trust from those who have come to fear government officials (e.g. through civil society, NGOs, the health professions, international alliances).

While such non-governmental functions are extremely valuable, they should never be regarded as an excuse for governments to avoid fulfilling their own responsibilities. These include setting policies and standards, providing resources, maintaining a peaceful and humane environment and ensuring efficiency and equity in the operation of state programmes that respond to the health needs of migrants and refugees in both short and long terms, as well as contributing politically and financially to international programmes that address both the causes and consequences of migration.

The M8 Alliance is an example of a group working at the international level that is, among other activities, promoting attention to the three inter-related issues of migration, health and climate change. Discussions on these issues have been featured in the World Health Summit annual meetings in Berlin and the first regional meeting of the World Health Summit in North America, which was held in Montreal in 2017. The first Expert Meeting of the M8 Alliance, held in Rome on 23–24 June 2017, whose discussions are integrated in this paper, focused on migrants’ and refugees’ health.

#### Education and training

Ensuring that the health needs of migrants and refugees are adequately met requires education and training, not only for the front-line workers who deal directly with people in transit and on reception and those health professionals who will encounter migrants and refugees in the ongoing delivery of services, but also for a wide range of other professionals who will come into contact with them including social, welfare, legal and employment bureau staff and interpreters, as well as policy makers. The range of education and training available must encompass not only knowledge of specific health issues that may be atypical of the host country’s population [including particular communicable diseases and the results of physical and psychological traumas from conflict and unsafe passage], but also of rights, cultural issues and inclusive approaches in attitude, language, policy and practice [126, 127]. Those treating special groups like trafficked persons also need focused guidance and training [128].

Facilities and courses that provide training in migrant and refugee health can be found in many countries, including Australia [129], Canada [130] and the USA [131]. In Europe, in addition to education and training available at the national level, at the regional level, there are training modules provided under the EC CARE programme. These address diverse aspects of the health of migrants and refugees, including health promotion and health care, reducing the burden of chronic non-communicable diseases and early detection of these diseases, communicable diseases surveillance and response, mental health, reproductive, cultural construction of health problems, confronting stereotypes, intercultural competences, violence and understanding migration [132]. WHO-EURO operated its first Summer School on Refugee and Migrant Health as an intensive 5-day course, ‘Managing the public health aspects of migration’, in Italy in July 2017 [133]. It aimed to improve participants’ knowledge and understanding of the main health issues and needs of refugees and migrants and of the broader public health and health-system implications of large-scale migration in origin, transit and destination countries.

Culture has an extremely important role to play in health in general [134], and responding to the health needs of migrants and refuges requires that health workers and their institutions develop ‘cultural competence’. This is defined as a set of congruent behaviours, attitudes and policies that come together in a system or agency or among professionals and enabling effective working in cross-cultural situations [135]. In the context of health care, it involves understanding and appropriately responding to the unique combination of cultural variables—including ability, age, beliefs, ethnicity, experience, gender, gender identity, linguistic background, national origin, race, religion, sexual orientation and socioeconomic status—that the professional and client/patient bring to interactions [136, 137]. The complexity of cultural sensitivity in clinical history and presentation must also be addressed. It is important to teach an approach that moves away from stereotypes (which increase negative attitudes to migrants). Also, allowance must be made for the fact that it takes time to think in a complex way, with a gap from collecting information to making sense of and interpreting it, and further time is additionally needed for development of a culturally sensitive treatment plan.

Furthermore, achieving meaningful cultural competence in health care requires more than provision of in-service training of health workers. It needs to involve systemic initiatives that can be developed around the idea of ‘cultural safety’—for example drawing on the Maori experience in New Zealand [138, 139]—and education in ‘transnational competence’ to address structural discrimination and avoid cultural blindness.

Koehn has discussed the importance of ‘transnational competence’ as a step beyond ‘cultural competence’, to prepare health workers for transnational medical encounters, emphasising five core skill domains (analytic, emotional, creative, communicative and functional) for migration health and the medical-school curriculum [140, 141].

Doctors, nurses and other health professionals among arriving migrants and refugees often face considerable barriers to being able to practice in the host country, including issues of language capacity and recognition of qualifications. This represents a considerable loss of potential—for the individuals themselves, who are often forced to take up less skilled and remunerative occupations; for the host countries, which are often experiencing a shortage of trained health professionals; and for the migrant and immigrant community, which would benefit from services provided by professionals who are closely familiar with their language, background and culture. A number of programmes and courses are attempting to bridge this divide—for example those of the International Labour Organization (ILO) [142] and some European countries [143, 144], but much more could be done to expand their scope and impact and to develop policies that systematically reduce the barriers.

There is a need for training in the treatment of children traumatised by events such as conflicts, disasters, arduous and dangerous journeys, confinement and poor living conditions. The Children and War Foundation has developed three manuals: Teaching Recovery Techniques, Writing for Recovery Manual and a Grief Manual, to help fill the gap in existing measures available to professionals [145]. Available free, these evidence-based resources have been developed by groups of experts and their effectiveness demonstrated in field projects in Africa, Asia, Europe and the Middle East supported by the Foundation, which also conducts training of interveners based on the manuals.

An example of an innovative new education programme that addresses a number of dimensions of the challenges of migrants’ and refugees’ health is the ‘Bachelor of Open Studies in Precision Public Health’ at the Institute of Global Health at the University of Geneva. This is being developed to begin in 2018 and will be based around 60 Massive Open Online Courses (MOOCs) offered in two tracks, plus a field research project. It will comprise equal amounts of public health and data science. ‘Precision public health’ is a transformative concept [146] which aims to bring the right interventions on health care and prevention, at the right time, to the right segments of the population. It converges cutting-edge digital technologies with innovations to better target evidence-informed interventions to the health needs of populations. As a relatively inexpensive (c. US$2000 per year) programme operated online through partnerships with universities in other countries, the degree course will be accessible to a very wide range of entrants who would not be able to undertake on-site education in a degree programme in Switzerland, including those blocked in a refugee camp anywhere in the world and migrants and refugees with limited resources.

### Case studies

Against a background in which there was inadequate knowledge of and attention to the specific health needs of migrants and refugees and great variability in policy, practice and social attitudes towards them, the unprecedented escalation in migrant and refugee numbers in recent years has presented major challenges, including from economic, social and health perspectives. This section provides insights into how a number of key actors have responded to the health challenges, including examples at regional (EU), country (Germany, Iran, Italy, South Africa, Turkey) and provincial (Quebec, Canada) levels.

#### Europe: the European Union and WHO region

The EU has experienced an unprecedented influx of refugees, asylum seekers and other migrants, with 1.5 million people arriving in 2015 alone [more than double the previous year], fleeing countries affected by war, conflict or economic crisis. This prompted the EU to identify the phenomenon as a crisis and ‘as the immediate priority of action in the EU’ [147]. Between 2000 and 2015, Europe hosted the second largest number of international migrants (20 million, 1.3 million per year) after Asia [148].

Arrivals have predominantly been initially through the sea borders of Greece and Italy [increasingly the latter as the Balkan route has been closed down] [149] but with those arriving often aspiring to reach northern Europe. While most refugees, asylum seekers and migrants are usually young adults, migrant populations recently arriving in the European Region have included many elderly and disabled persons, as well as an increasing number of minors, many of whom are unaccompanied children. Gender differences in health status are seen. Women, including pregnant women, comprise half of all refugees, asylum seekers and migrants and are often disproportionately represented in vulnerable groups, such as victims of gender-based violence, human trafficking and sexual exploitation. Risk factors that affect men in particular include exposure to accidents, physical stress and other work-related health hazards. Evidence also suggests higher mental distress among refugee and migrant populations, with increased risk for women, older people and those who have experienced trauma, and further risk caused by lack of social support and increased stress after migration [150].

Efforts have been made to reform the Common European Asylum System to create a fairer, more efficient and more sustainable system for allocating asylum applications among Member States, while retaining the basic principle of the Dublin Regulation [151]. This requires that asylum seekers should, unless they have family elsewhere, apply for asylum in the first country they enter. The reforms aim to ensure no Member State is left with a disproportionate pressure on its asylum system [152, 153]. The OECD estimates that the cost for processing and accommodating asylum seekers is around €10,000 per application for the first year [154].

A Communication from the EC on ‘A European Agenda on Migration’ in 2015 was structured around four pillars to manage migration better: reducing the incentives for irregular migration; border management—saving lives and securing external borders; Europe’s duty to protect: a strong common asylum policy; and a new policy on legal migration [155]. The Communication promised the mobilisation of an additional €60 million in emergency funding, including to support the reception and capacity to provide health care to migrants in the Member States under particular pressure. It identified the European Regional Development Fund and European Social Fund as potential sources of support for, among other areas, investments in social inclusion and services. However, a major and contested element of the EU responses to the refugee situation has been its budgetary response [156].

The EU doubled its budget for the refugee crisis to €10.1 billion in 2015–2016 [157]. However, at the World Humanitarian Summit in May 2016, Germany’s Minister for Development Gerd Müller criticised EU’s mechanisms for responding to refugee crisis as not fit for purpose. Germany argued for diverting a further 10% (c. €10 billion) of the EU budget towards dealing with the refugee crisis, after a lack of joined-up thinking exacerbated the challenges posed by irregular migration to Europe [158]. Amid mounting tensions over the costs of coping with arriving refugees [159, 160], by 2017, the total EU budget for refugees had risen to €17.7 billion, including €9.2 billion for humanitarian aid inside and outside the EU and €0.7 billion for support to livelihood opportunities, health and education for refugees and mobility policy outside the EU [161].

The development-led approach to forced displacement, adopted by the EC in April 2016, focuses on working with host governments, at the national and local level, towards the gradual socio-economic inclusion of refugees and internally displaced persons, aiming to harness their productive capacities by helping them to access education, housing, land, livelihoods and services [162]. The EU’s objective is to strengthen the resilience and self-reliance of both the displaced and their host communities through a multi-actor approach from the outset of displacement crises.

Within this highly pressured regional context, and with relatively small amounts of EU money being allocated for emergency health support, individual EU Member States have retained responsibility for health and face a pressing need to address, among other issues, the health needs of the migrants and refugees on reception, during transit and at their destinations [163, 164].

Unfortunately, as observed by Roberts et al. [165], in practice, the health needs of the migrants and refugees on reception and during transit have often met with a lamentably inadequate response, characterised by lack of access to even basic primary health care, including maternal and child health services, and lack of the continuity of care on which the health of those with NCDs depends. Concerns have also been raised about the risk of sexual and gender-based violence, while children and young people are being separated from their families and left with limited protection. Governments in several countries further restricted the already limited entitlements of undocumented migrants, leaving the task of caring for them to civil society, including new volunteer groups as well as established international humanitarian agencies such as Médecins du Monde, Médecins Sans Frontières, Save the Children and the Red Cross.

A systematic review examined undocumented migrants’ entitlements and barriers to health care as a major public health challenge for the EU [166, 167]. Infectious diseases, chronic illnesses, mental disorders, maternal-child conditions, dental issues, acute illnesses and injuries were the most common pathologies presented. However, in most cases across Europe, undocumented migrants have access only to emergency care. Even in countries where they are fully entitled to health care, formal and informal barriers hinder them from accessing or feeling entitled to this right. Socio-cultural barriers, such as language and communication problems, lack of formal and informal social and health care networks and lack of knowledge about the health care system and about informal networks of health care professionals are all common impediments. From the health care providers’ perspective, there can be difficulties in providing appropriate care and in dealing with cultural and language barriers and false identification. There were few available examples of policies and best practices aimed at overcoming barriers in the delivery of health care to undocumented migrants. It was concluded that better communication strategies and dedicated communication services were needed to help address the inequalities in access to health care services, that the definition and provision of specific training focused on undocumented migrants’ health needs were desirable and that research should be strengthened.

The deficiencies have highlighted the critical need for an effective policy response [168] nationally and internationally, both to meet the immediate and longer term needs of those who have made their way to Europe and to address the causes of leaving their homes and the conditions they face in the countries immediately adjacent to those where they lived, which bear the brunt of refugees. In developing the response, it is important that a key lesson of past migrations is recognised—restricting health care is economically counterproductive [169].

A high-level meeting of WHO-EURO on Refugee and Migrant Health was held in Rome, Italy, in November 2015 [170]. All European countries agreed [171] on the urgent need to develop a European framework for collaborative action on refugee and migrant health, based on the principle of solidarity and humanity. It was noted that, while most Member States of the European Region have the capability to respond to the public health challenges associated with migration, they may still require better preparedness, greater capacity for rapid humanitarian response and increased technical assistance. The events in 2015 had pushed the capacity of individual countries to the limit and development of resilience to sustained migration was needed. This was an opportunity not only to deal with short-term needs but also to strengthen public health and health systems in the longer term.

A draft European strategy and action plan on refugee and migrant health [172] accompanied by a resolution [173] was submitted to the next Regional Committee for Europe meeting in September 2016 and adopted [174]. This called for action relating to nine strategic areas covering establishing a framework for collaborative action; advocating for the right to health of refugees, asylum seekers and migrants; addressing the social determinants of health; achieving public health preparedness and ensuring an effective response; strengthening health systems and their resilience; preventing communicable diseases; preventing and reducing the risks posed by non-communicable diseases; ensuring ethical and effective health screening and assessment; and improving health information and communication. WHO-EURO will coordinate the implementation of the action plan and report on progress to the Regional Committee in 2018.

#### Germany

Protection of migrants and refugees has become a cornerstone of present-day German policies, as well as the fight against the root causes of refugees and cooperation with African and other countries. Germany is the largest host of refugees in the EU, spending more than €20 billion domestically [175] on refugees in 2016 and is the EU’s largest donor to external refugee support [176]. Official Development Assistance from Germany increased by more than a third in 2016, due to the scaling up of its overall aid programme as well the doubling of in-donor refugee costs compared to 2015 [177, 178].

At the political level, Germany placed health on top of the issues of its G20 presidency in 2017, although it did not deal explicitly with the health of migrants and refugees [179, 180]. A Lancet Series, launched just before the Hamburg G20 Summit, examined Germany’s health system and the country’s growing financial and political interest and involvement in global health [181].

Nationally, Germany’s health response to its large influx of refugees has included shifting policies, reallocating health care resources and allocating new resources.

Health is a Federal responsibility in Germany, but while the Federal Ministry sets the conditions for medical care, the further specific setup, organisation and financing of individual medical services is the responsibility of legally designated self-governing bodies within the health care system, including the Ministries of Health of the 16 Federal States [182].

Cities have a critical role to play in responding to influxes of migrants and refugees, and in practice, very important early aspects of Germany’s response, including in the health field, came at the city level. A study of Germany’s 15 largest cities hosting refugees noted that the current framework for allocating funding and expenditures across federal, state and city governments imposes uneven burdens on city-states and large cities. Nevertheless, cities such as Berlin and Hamburg, which have received the largest numbers of migrants, have shown a remarkable ability to innovate in the face of crisis, including an expanded role of civil society, unifying the delivery of services, the use of technology to engage community participation and the rapid building of non-traditional housing. In 2015, Hamburg’s expenditure on health care for refugees in reception centres totalled €58.6 million. The city-states have also provided an early warning system for the federal republic and helped to reform restrictive federal laws to be more responsive to local needs and circumstances [183].

Berlin, with a population of 3.5 million, received about 80,000 asylum seekers in 2015, who were initially housed in tent camps. Charité-Universitätsmedizin Berlin, which is wholly owned by the Federal State of Berlin, decided in September 2015 to supply health services to refugees where they were and asked its doctors to help. They rapidly established a number of refugee clinics and, in February 2016, the first central point nationwide for psychiatrically ill or traumatised refugees. In March 2016, they began mandatory initial medical screening, including TB screening and vaccinations. An initial catch-up vaccination programme was started using a shuttle bus bringing in refugees and was succeeded by a vaccination bus to reach out to them in November 2016. By this time, the 42 doctors and nurses and many volunteers involved in the provision of these health services had seen over 64,000 patients. One vital aspect was that on-line interpretation teams were established, with over 50 languages available within 1–2 min.

In the period September 2015–February 2016, demographic data on those asylum seekers treated in Berlin showed two peaks, corresponding to children and young adults, with 39% coming from Syria, 31% from Afghanistan and 18% from Iraq. Most (65%) asylum seekers came to clinics because of infectious diseases, 70% of which were upper airway infections (which was expected, as it was winter). NCDs accounted for 15% of diagnoses, while mental and behavioural disorders accounted for a very small fraction [2%] of cases presenting in this early period. This was a surprise, as a very low threshold had been set for access to psychiatrists for the refugees and it was expected to see many more patients. Of those presenting, 95% needed further treatment.

The greatest health challenge related to those refugees and migrants was not in camps with social workers present, but in the city, as these people received less help.

Important lessons from the experience in Berlin included that significant difficulties of the public service and public health in Berlin in treating migrants and refugees came under control with the support of hospitals and many volunteers; systematic mandatory initial medical screening of refugees started late in Berlin, resulting in missed opportunities for preventive health care prior to March 2016; the catch-up vaccination programme, initially by bus shuttle and subsequently by vaccination bus, worked effectively; establishing a central point for psychiatrically ill or traumatised refugees, with low-threshold access, was very valuable; and access to rapidly available interpreting services was highly useful.

#### Iran

Iran is host to one of the world’s largest and most long-standing refugee populations. There are nearly 1 million documented refugees in Iran, the vast majority being from Afghanistan, plus c. 30,000 from Iraq, and there are approximately 1.5 million undocumented migrants plus 620,000 Afghans with passports and Iranian visas living in Iran [184]. Most refugees reside in urban areas, with less than 3% living in 20 settlements. The majority of the refugee population are second and third generation and under 24 years old. Iran has placed much emphasis on ensuring the education of refugees, and the literacy rate among Afghan refugees rose from 6% in 1979 to 60% in 2013. Since May 2016, refugee students receive the same benefits as Iranian students regarding registration and educational services. Iran is assisted by UNHCR in its regional plan for Afghan refugees in South-West Asia [185, 186].

In 1976, Iran acceded to the 1951 Refugee Convention and its protocol [1967] with reservations on articles relating to wage-earning employment, public relief, labour legislation, social security and freedom of movement. The universal right to social security is enshrined in the country’s Constitution, which asserts that, in case of accidents and emergencies, everyone has the right to health and medical treatments through insurance or other means. All children are given rights to education and other services. Refugees are given health insurance for accidents and illness. The government has started gradual registration of undocumented children, of whom about 50,000 are already enrolled in schools.

Maternal mortality is quite high in Iran [187], one factor being a lack of skilled birth attendants. Iran is collaborating with the UN Population Fund (UNFPA) and Italy in a project to improve maternal health in Afghanistan through an Iranian-Afghan academic collaboration to build the capacity of the midwifery department at Kabul Medical University, to improve maternal health for Afghan refugees in Iran by developing an Afghan-friendly maternity ward at a Tehran hospital and to qualify 50 Afghan refugee women as diploma-level midwives and 10 at Masters level, using the Afghan midwifery curriculum to enhance repatriation and reintegration prospects in Afghanistan [188, 189].

The model of health system governance is unique in Iran, with the Ministry of Health and Medical Education not only overseeing medical education but delegating implementation of health policy to medical universities across the country [190]. Iran aims to reach universal health coverage [191] by 2025 for all residents in the country, irrespective of status, through implementation of the 2014 Health Transformation Plan or Health Sector Evolution Plan [192]. The number of people with health insurance has increased rapidly, rising from 68% in 2007 to 92% in 2015.

Iran offers health insurance to refugees [193]. Documented migrants and refugees are covered by the Universal Public Health Insurance scheme (Salamat Health Insurance) [194]. Undocumented migrants are assisted by NGOs, including Mahak Charity (helping children with cancer, with a hospital in the north of Tehran), Behnam Daheshpour Charity (helping patients in need of cancer care) and Zanjireh Omid (treating underprivileged children suffering from cardiac, orthopaedic and reconstructive diseases)*.* Refugees’ free access to primary health care is not restricted. Examples of free primary health care services for refugees include vaccinations, antenatal care, maternal and child health, family planning, health promotion activities, nutrition consultation, psychology consultation and treatment of HIV conditions and TB, and there are many health promotion classes.

Community-based rehabilitation (CBR) services for refugees are provided in Iran through partnerships between the State Welfare Organization of Iran, Bureau for Aliens and Foreign Immigrants Affairs and UNHCR. Access to all rehabilitation services is provided in public or private centres, as for Iranian citizens. Access to CBR services for refugees with disabilities includes surgery to reduce disability, medical referral and treatment (including the cost of doctor visits, medication, testing and para-clinical services), referrals to rehabilitation centres to receive physical therapy, occupational therapy, speech therapy, audiology, optometry, psychology and rehabilitation counselling. One thousand one hundred sixty refugees with disabilities used the CBR services in 2016.

Challenges experienced by Iran in providing health insurance and services for refugees include the low social and economic status of refugees, the difficulty of funding provision for large numbers of refugees with diseases that are expensive to treat, the lack of culture of risk aversion in refugees who do not welcome health insurance, the presence of a large numbers of undocumented migrants in Iran, the lack of familiarity with health care service and the avoidance of utilising services due to fear of being deported to Afghanistan.

Social attitudes towards migrants in Iran vary. Generally, migrants are welcomed in urban areas where they mostly settle and where many Afghan refugees make a large labour contribution, but they have been less well regarded in some provinces where there are few refugees.

#### Italy

Italy’s geographic position and c. 7500 km of coastline have resulted in it being at the forefront of arrivals of migrants and refugees by sea. According to UNHCR [195], there were 95,213 new arrivals in 2017 up to the end of July, a 2% increase on the same period in 2016 [93,774]. Of these 2017 arrivals, 17% originated from Nigeria, followed by Bangladesh (9%), Guinea (9%), Côte d’Ivoire (8%), Mali (6%), The Gambia (6%), Senegal (6%), Eritrea (6%), Sudan (5%) and Morocco (5%). Nigeria remained the most common country of origin of sea arrivals in Italy in 2017, as in the same period the previous year, while Bangladesh and Morocco did not feature among the main nationalities of arrivals in the first 7 months of 2016. While over 11,000 Eritreans reached Italian shores in January–July 2016, 5325 arrived by sea in January–July 2017. Men accounted for three quarters of the 2017 group, and the point of departure for 96% of the group was Libya.

**Fig. 7:**
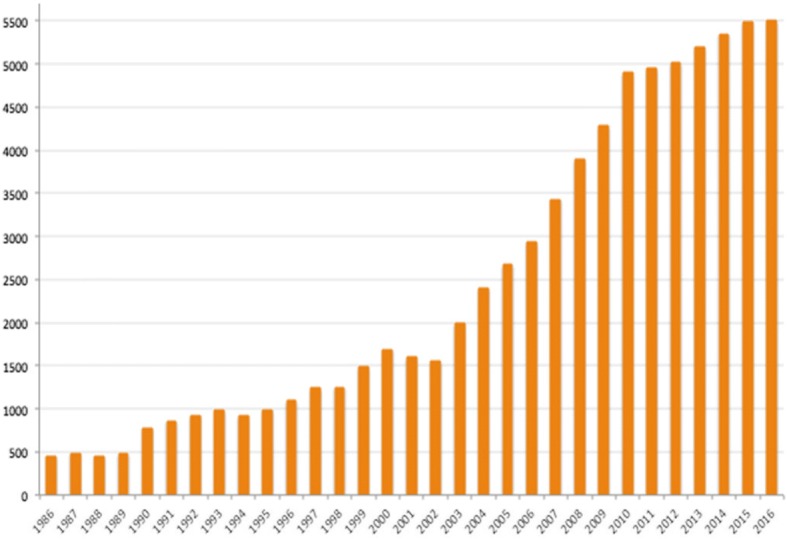
Immigration in Italy: annual numbers of legal immigrants (× 1000), 1986–2016. Source: data from the Istituto Nazionale di Statistica and Italian Ministry of the Interior, compiled by S. Geraci, SIMM, 2017

There has been a tenfold increase in immigrants to the country since the 1980s (Fig. 7).

Italy has a long tradition of outward migration of its own population, which saw 27 million citizens emigrate between the mid-nineteenth and mid-twentieth centuries. The country continues to experience an outward flow of its citizens—especially highly skilled young people [196]. Against this background, Italy’s legal system has historically been generally favourable to incoming migrants. However, there are distinct regional variations between the south and north, and the recent large influxes have strained the Italian economy and created tension within the EU.

The 1948 Italian Constitution speaks of individual rather than citizen rights, which is reflected in an inclusive health policy that guarantees free medical care to the indigent [197]. Italian law forbids reporting to the police undocumented migrants who have received a health service.

An important and effective advocate for Italy’s inclusive laws has been the Italian Society of Migration Medicine (SIMM) [198], which was established in 1990. With about 500 active members, SIMM serves both as a scientific society and as a national policy network for exchanging experiences, data, scientific evidence and considerations on health policy, including at the local level, relating to migrants’ right to health care.

The current Italian regulations on the rights of migrants to health care services date from a comprehensive 1998 law [199] and successive regulatory provisions, including one in 2000 from the Ministry of Health which rules that asylum seekers benefit from free health services on the basis of a self-declaration of destitution, so that they are treated under the same rules as unemployed Italian citizens—although the practice is very different throughout the country [200]. Another Ministry of Health decree provides guidelines [201] for the planning of care and rehabilitation interventions and for the treatment of psychological disorders of refugees that have suffered torture, rape or other forms of psychological, physical or sexual violence.

Broadly, Italy’s policies on migrants’ health care afford the possibility of health protection and health assistance, provision of hospital and outpatient care whether for emergency or continuous treatment, covering essential illness conditions, preventive medicine and rehabilitation. They offer particular protection for women and children and special attention to infectious diseases and international prophylaxis.

For the last decade, Italy has been positively engaged in almost all the recommendations of the 2008 WHA resolution on the health of migrants [202] which call on Member States to promote migrant-sensitive health policies; promote equitable access to health promotion, disease prevention and care for migrants, subject to national laws and practice, without discrimination on the basis of gender, age, religion, nationality or race; establish health information systems in order to assess and analyse trends in migrants’ health, disaggregating health information by relevant categories; devise mechanisms for improving the health of all populations, including migrants, in particular through identifying and filling gaps in health service delivery; gather, document and share information and best practices for meeting migrants’ health needs in countries of origin or return, transit and destination; raise health service providers’ and professionals’ cultural and gender sensitivity to migrants’ health issues; train health professionals to deal with the health issues associated with population movements; promote bilateral and multilateral cooperation on migrants’ health among countries involved in the whole migratory process; and contribute to the reduction of the global deficit of health professionals and its consequences on the sustainability of health systems and the attainment of the Millennium Development Goals.

However, there have been conflicts between the Italian state and regional authorities, with persisting differences in interpretation and practical application [203]. The state has exclusive competence for immigration and asylum policy, while health care policy is a competitive competence between the state and regions. One contentious issue has been the law forbidding reporting to the authorities of undocumented aliens receiving health services, which has been in force since 2009 despite attempts to overturn it.

Barriers encountered by migrants and refugees in accessing health services in Italy include bureaucratic/administrative, economical/financial, linguistic/cultural organisational and psychological. Critical situations for migrant health include access to reproductive, occupational and mental health services, with discriminatory practices by some health service providers. Abortion rates [204] were three times higher for migrant women than for Italian women in 2013, and the occupational risks [205] among migrant workers in Italy are significantly higher.

A study, undertaken by Anna Massetti of Sapienza University, Rome, of the health profile of migrants living around Rome as asylum seekers, refugees or undocumented migrants, examined the most frequent diagnoses, social determinants and access to care of asylum seekers at the CARA Centre (an inpatient facility 30 km from Rome) and at an outpatient clinic for foreigners at University Hospital Policlinico Umberto I (UHPU) in Rome that deals with recently arrived asylum seekers and also long-standing undocumented migrants. This was a cross-sectional retrospective study with a 1-year observation period, in which socio-demographic data were collected and diagnoses performed according to ICD-10 [206] (psychiatric disorders were not included in CARA).

The CARA Centre saw 792 asylum seekers from 43 different countries, including 80% male and nearly 9% children, with a mean age of 27.4 (SD ± 9.4) years. The main countries of origin were Pakistan (13.1%), Nigeria (12.4%), Afghanistan (10.9%) and Eritrea (7.6%). The mean length of stay in CARA was 268 days and 2843 diagnoses were recorded. In 192 cases (6.7%), asylum seekers were referred to hospitals for a second-level examination and 50 (26%) were hospitalised for surgical (12), medical (12), psychiatric (9) and gynaecological/obstetrical (17) reasons. There were six cases (0.2% prevalence) of active TB. In voluntary screening for infectious diseases, 347/729 adults (47.6%) accepted screening, with a profile of 77.8% male, 35.8% from West Africa, 24.2% from South Asia and 21.9% from East Africa. Findings included syphilis (all male, 72% from Africa), HIV (50% male, all from Africa), hepatitis C virus (HCV, 82% male, 54.5% from Asia), hepatitis B virus-infected (HBV, 85% male, 75.7% from Africa) and HBV-exposed (86% male, 67% from Africa).

At the UHPU outpatient clinic, the total of 939 patients comprised 188 asylum seekers and 751 long-standing undocumented migrants from 62 different countries. Patients generally used the facility as a family practice for general medicine needs. Among the asylum seekers (mean age 22.3 years), all with HBV or *Treponema* infection were male from West Africa and two out of seven HBV antibody-positive patients had active chronic infection and were referred to a hepatitis centre in order to start treatment. There were 72 unaccompanied minors in the asylum seeker group (age range 13–17 years, only one female), with extremely diverse diagnoses.

The study concluded that, with an extremely heterogeneous population and range of health issues, guidelines for screening are needed: there were no guidelines available in Italy for medical examination and/or infectious diseases screening of asylum seekers in place at the time of the study. An immunisation policy among asylum seekers needs to be defined for HBV and other infections. Barriers to acceptance of preventive care/screening included social diversity, language barriers and cultural beliefs. Treatment was best accepted when patients were accompanied, but only if by persons understanding its importance.

Among the undocumented migrants (60% males with mean age 38.6 years, 40% females with mean age 37.4 years), predominant health problems included gastrointestinal, musculoskeletal, cardiovascular, endocrine, nutritional and metabolic diseases.

Overall lessons from Italy include the positive approach of inclusive health policies and good education and training of health professionals and the benefits of an active policy network undertaking organised and effective advocacy action within the framework of the new paradigm of global health. Ongoing challenges include the lack of coherent governance, with disparate application of rules in different territories, lack of adequate social protection policies for vulnerable groups and difficulties in planning and implementing cross-sectoral policies.

#### South Africa

South Africa has a long tradition of inward migration, with many economic migrants, especially from other African countries but also India, China and Pakistan, being attracted by opportunities for work in industries such as mining and particularly being concentrated in Gauteng Province. However, there is a lack of precise data on the number of migrants in the country [207]. The 2016 Community Survey by Statistics South Africa reported that the total population of approximately 55.7 million included 1.6 million migrants, which was less than the 2.1 million recorded in the 2011 census. However, the UN’s Department of Economic and Social Affairs estimated that in 2015 there were over 3.14 million international migrants living in South Africa. The apparent decline and discrepancies may be a result of foreign-born people fearing to disclose their origin [208]. The number of asylum seekers living in South Africa was put at 1.1 million in 2015, but that and other recent annual figures have been heavily disputed [209, 210].

Xenophobia and waves of violence against immigrants and also against domestic migrants and ethnic, religious, and political minorities have been seen in recent years, including riots, rapes, murders and looting and burning of property [211, 212]. A 2015 UNHCR report [213] notes that the xenophobia has deep social, political and economic roots that are closely intertwined and that long-term solutions will require addressing xenophobic sentiments and practices within government institutions together with social campaigns and legislative reform, as well as a judicial response to counter the culture of impunity that enables perpetrators and related parties to profit from xenophobic violence, scapegoating and other forms of exclusion. There is a need for a clearer understanding of the drivers of violence [214, 215], and an IOM report [216] observed that, while the legacy of previous regimes and continued institutional discrimination provide broad structural and historical explanations, the emergence of xenophobic violence is typically rooted in the micro-politics of township life, with local sentiment being manipulated and false information disseminated.

Against this background, the promotion of access to health services for migrants in South Africa is a contested political topic. A nuanced and complex picture emerges from studying local and international migrants’ access to care services.

Legally, there is a Constitutional right to health in South Africa, which has been clarified and confirmed in jurisprudence—including access to anti-retroviral therapy for HIV. Both legal and undocumented residents have the right to primary care, with the same entitlements being applied to refugees and asylum seekers as for all South African citizens.

The history of HIV/AIDS in South Africa has been complex [217]. The massive health challenge created a potential basis for social inclusion and justice, but the Government’s response, marked for many years by denial, lack of political will and poor implementation of policies and programmes and a dysfunctional health service, led to a situation of anomie, and there was massive overload and some health workers showed abusive attitudes to patients. Migrant labour was one of the significant vectors for the growth of the HIV and associated TB epidemics.

In practice, South African health care professionals have routinely denied health care and treatment to many asylum seekers, refugees and migrants, with foreign-born residents encountering systematic discrimination in obtaining basic care [218, 219], and, in addition to many other factors, language barriers have played a significant role in poor treatment [220]. There is evidence that barriers to accessing health care have particularly affected migrant workers in mining and commercial farming. A healthy migrant effect has been observed, with physically healthy individuals migrating to undertake labour, but migrant women in general are particularly vulnerable, including to HIV infection, exploitation and gender-based violence [221]. Little research has been undertaken to explore access by forced migrants [refugees and asylum seekers] in South Africa to public health care, including services for mental health and psychosocial support, but evidence suggests that policies designed to protect the health and psychosocial rights of urban forced migrants are not being effectively implemented, for example in Johannesburg [222]. Practices are highly variable across provinces, facilities and practitioners. For example, significant barriers to access have been particularly documented in Gauteng, including xenophobic attitudes and behaviours of providers, provincial directives recommending up-front payment in contradiction to National Department of Health directives, abuse of patients and problems with language and ethnicity impacting on routine care.

In practice, civil society organisations and health activists play key roles in helping to provide or secure health services for migrants and refugees.

Concern about the living and working conditions of farm workers, including migrants, in the Western Cape has prompted a number of studies [223, 224] that have highlighted problems including human rights abuses, inadequate and insanitary housing, exposure to dangerous pesticides, lack of access to safety equipment, sanitation and drinking water while working, rampant alcohol abuse, domestic violence, inadequate treatment of injuries occurring while working and failure to provide sick leave or maternity leave.

A study [225] of migrants’ access to health care in De Doorns, Western Cape, undertook a community survey of 807 migrant and local households, in which half came from South Africa, and around a quarter each from Lesotho and Zimbabwe. The majority of people has access to care, but the percentage was higher [88%] among South Africans than among migrants [77%]. Services were used more by females [89%] than by males [74%], with the conditions for which treatment was sought including [from most to least] HIV, sick child, hypertension, TB, pregnancy and diabetes. Non-South Africans spent significantly longer at health facilities than South Africans, expressed lower overall levels of satisfaction and considered staff less friendly. There was a major difference in the quality of child care, with most South African children being placed in crèches, while most international migrants’ children were placed with a ‘day mother’ with very poor facilities.

In conclusion, migration in South Africa is extensive, rather poorly documented, contested and exploited politically. Access to health care, while in principle provided for in laws and regulations, is extremely variable in practice and heavily impacted by systemic factors, varying openness of practitioners and managers and xenophobic attitudes by health professionals at all levels. Lessons include that history, inequality, politics and context matter, but amidst an ever-evolving interplay of population, place and power, it is possible and necessary for health to create a space to exercise evidence-based and rights-based agency to influence politics, policy and practice.

#### Turkey

Turkey has the largest refugee population of any country in the world—the figure standing at around 2.5 million in 2015 and rising to around 3.5 million in 2017, with most of the refugees coming from the Syrian Arab Republic and Iraq [226, 227]. Turkey carries a large financial burden in coping with this challenge, contributing 0.75% of its gross national income (GNI)—the largest proportional contribution of GNI to humanitarian assistance of any country in the world [228].

While the earlier practice was to disperse most refugees across Turkey’s 81 provinces [229], since 2012, an increasing number have been housed in Temporary Accommodation Centres established under the control of the government’s Disaster and Emergency Management Authority [AFAD] [230]. Nevertheless, in mid-2017, out of the more than 3 million registered Syrian refugees in the country, 246,720 people were hosted in 23 camps run by AFAD, where refugees have access to shelter, health, education, food and social activities with international assistance. Those remaining outside of camp settings [the largest numbers of which were in Istanbul, Sanliurfa, Hatay and Gaziantep] had limited access to basic services.

The EC has been providing humanitarian assistance to vulnerable refugees, particularly to those living outside of camps. The EU and its Member States are funding the ‘EU Facility for Refugees in Turkey’, providing €3 billion for the needs of refugees and host communities through humanitarian and development assistance in 2016 and 2017. In addition, the EC provided €348 million through the Emergency Social Safety Net (ESSN), a debit card-based social assistance scheme to allow up to 1.3 million refugees to cover their basic daily needs.

The massive and rapid increase in Syrian refugees arriving and the prognosis that the Syrian crisis will not be ended soon has necessitated that the government and civil society switch gears from policies driven by concerns of extending emergency humanitarian assistance and temporary protection to ones focusing on the long term, to facilitate the possible eventual incorporation of the refugees into Turkish society. Turkey should not have to bear the cost of this policy transformation alone. Protecting and caring for refugees is an international responsibility [231] while Turkey must adjust to the legal, economic and political challenges [232].

Health support for Syrian refugees living inside and outside Temporary Accommodation Centres has been the responsibility of the Turkish Ministry of Health since the first refugees began to arrive in April 2011. Major health issues seen in the refugees include injured and wounded people, unvaccinated children, uncontrolled chronic disorders, refugee patients in a critical condition requiring intensive care and untreated infectious diseases (acute gastroenteritis, TB, respiratory tract infections, sexually transmitted diseases, leishmaniasis, measles, polio, hepatitis and HIV) [233]. Overall, the level of infectious diseases was less than expected, but the presence of polio was a particular challenge in the context of the Global Polio Eradication Initiative and a mop-up vaccination programme was instituted.

Data on the distribution of infectious diseases among refugees according to year indicated increases in diarrhoea and respiratory tract infections from 2012 to 2016. This may be related to the poor living conditions outside the Temporary Accommodation Centres. In the refugee camps, living conditions are regulated and there are clean water sources, control of waste and surveillance for diseases such as leishmaniasis, malaria, TB and gastrointestinal infections, with provision of first-line health services, triage to hospital and intensive care units, services for reproductive and mother and child health and polio and measles vaccinations for under-15-year-old children. All refugee children are included in the Turkish National Vaccination Programme.

Up to mid-2017, 1300 physicians and 700 nurses were trained and included in a training programme for the Refugee Health System, conducted by a multidisciplinary team and consisting of 6 weeks practical and 1 week theoretical education.

#### Cities and provinces: Montreal, Quebec

As already exemplified for the case of Germany, in federal and other decentralised systems, responsibility for health often devolves to the provincial and city levels, challenging the resources locally but also providing opportunities for speed and innovation in response to the needs of migrants and refugees.

Canada has a long history of receiving migrants and refugees [234], and most recently, this has included about 6600 Bhutanese refugees arriving in Canada in 2015 and more than 40,000 Syrian refugees between November 2015 and January 2017. In response to thousands of migrants who had been denied asylum in the USA crossing the border into Quebec, in July 2017, Canada opened the Montreal Olympic Stadium as a temporary shelter to host them. Manitoba and British Columbia have also both seen an influx of hundreds of asylum seekers [235].

Roles and responsibilities for health care services are shared between provincial and territorial governments and the federal government in Canada. The provincial and territorial governments are responsible for the management, organisation and delivery of health care services for their residents, while the federal government is responsible for setting and administering national standards for the health care system through the Canada Health Act, providing funding support for provincial and territorial health care services, supporting the delivery for health care services to specific groups [including some groups of refugee claimants] and providing other health-related functions. Canada’s Medicare is a publicly funded health care system operating through 13 provincial and territorial health care insurance plans, which ensures that all Canadian residents have reasonable access to medically necessary hospital and physician services without paying out-of-pocket [236].

Previous work has indicated that immigrants living in Quebec show a healthy migrant effect on arrival, but this diminishes over time, with one important contributing factor being the different access that migrants have to health coverage [237].

A particular challenge is how each province treats access to health care for uninsured migrants. In Quebec, a mixed methods research study [238] was undertaken by a multidisciplinary and multilingual team, aiming to recruit 400 migrants from the broad community and 500 from the medical clinic of Médecins sans Frontières. To date, 633 had been recruited, with an average residence time in Quebec of 5.6 years and a period without health insurance [once they lost any initial coverage] averaging 3.2 years. This group included people from Africa, Latin America and the Caribbean, Middle East, Asia, Europe and the USA, with an average age of 41 and two thirds being women. About 40% were ‘without status’ as immigrants (about half awaiting processing of applications for permanent residence on humanitarian grounds, the rest without any process in place).

It was observed that, in the absence of the ‘card’ needed for free access to health care in Quebec, uninsured people regularly pay three times the real cost of health treatment in what appears to be official policy. There is no municipal budget to cover the uninsured and limited community initiatives, so that it is left to professionals to voluntarily ‘fit them in’.

More of those ‘with status’ had higher education and better finances. Among those ‘without status’, there was higher food insecurity, fewer working for placement agencies and more living in sub-standard housing and more self-reporting relatively poor health status (which was substantially higher for both groups than for Canadian-born residents). While important health distress was higher among those with status than those without status, very important psychological distress was higher among those without status, as were feelings of being nervous, hopeless and severely depressed. Sources of distress revealed in discussions included discrimination in health and worries about access to health care, difficulties in finding work, problems with immigration, fear of consulting and of deportation if services were used and fear of being identified by the Canada Border Services Agency. Among the study population, 62% had not consulted a health professional in the previous year. The main first-line resources used by those who experienced ill health [just over half the group] were pharmacy (70%), *Médecins du* Monde (22%) and drop-in clinic (19%), while in urgent cases, 18% used emergency hospitals.

Among the study population, 70% had felt the need for health services but did not receive them and 27% had not obtained medication that was prescribed. Barriers reported were, first, financial (more important for those ‘without status’); second, fear (much more important for those ‘without status’); and third, not knowing where to go for help.

Practical goals that emerged as important from the preliminary results of the study included recognition of the need to provide information and training for public and community agencies that support uninsured migrants in accessing health, to provide tools to improve access to resources and to improve the integration of migrant heath issues into training curricula of health professionals (including epidemiology, medicine, nursing, public health, social work).

### Research challenges

Good quality evidence on the nature of health issues and the effectiveness of treatment approaches is essential for policy and practice, as illustrated by the work of the migrant health subgroup of the Campbell and Cochrane Equity Methods Group [239], which focuses on evidence-based migrant health, guidelines and migrant equity. However, as highlighted throughout this paper, there are many gaps in the present state of knowledge about the health of refugees and migrants and how best to attune health services to meet their needs. Other papers and articles address priorities for research on the health of forced migrants [240] and point to the need for research in different settings such as refugee camps [241] and arriving groups [242].

Studying the health of migrants and refugees poses particular challenges due to the mobility of these groups and additional complications including cultural, educational and linguistic diversity as well as legal status. These factors may serve to limit the usefulness of both traditional survey sampling methods and routine public health surveillance systems. A handbook of research methods appropriate for migration and health has been developed [243].

A study reported at the M8 Alliance Expert Meeting in Rome compared research findings from Italy and Sweden. A search of PubMed for research on migrants’ health in Italy yielded 301 reports, beginning in the mid-1960s, displaying a hyperbolic increase in the last 10 years, while a similar search for Sweden returned 172 reports, also showing an increase in the last 5 years.

There were substantial differences in results between the two countries. The research reported from Italy had a major focus on acute cases, dealing with epidemiology and prevention of infectious diseases, as well on traumatic issues. Study designs were cross-sectional. For Sweden, however, studies had a major focus on social vulnerabilities, psychosocial problems and disability pensions, with study designs being of the cohort type reflecting the need to follow people rather than only examine them at one point in time.

**Fig. 8:**
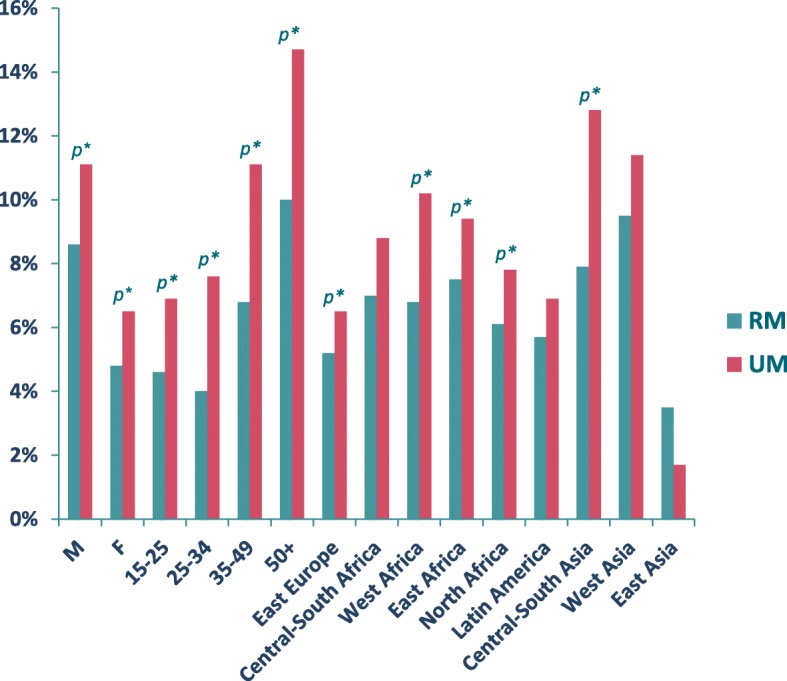
Rates of avoidable hospitalisation of undocumented (UM) and regular (RM) migrants. Source: Giuseppe La Torre, Sapienza University

Two case studies were undertaken. One examined avoidable hospitalisations in migrants in Sicily, which has become one of the migrant gateways to Europe. Undocumented migrants there are entitled to outpatient and inpatient services for urgent or essential care and to preventive services—in particular in the fields of maternal health, minors’ health, vaccination and prevention and treatment of infectious diseases. The study examined hospital discharge schedules from 2003 to 2013 and defined and identified hospitalisations which could have been prevented through effective primary care or preventive services. In a stratified analysis (Fig. 8), undocumented migrants showed an increased rate of avoidable hospitalisation compared to regular migrants, for all age groups and almost every region of migrant origin.

The second case study examined suicide among first-generation and second-generation immigrants in Sweden. This was a prospective, population-based cohort study of 4,034,728 individuals aged 16–50 years, followed from 2005 to 2010 and analysed for first-generation, intermediate-generation and second-generation immigrants compared with natives, controlled for socio-demographic factors, morbidity and labour market marginalisation. In the first generation, there was a lower suicide rate among migrants, but among intermediate- and second-generation migrants, the rate found was higher than that for the general population.

Drawing on the results of the two studies, which contrasted emergency with stable population health care, it was noted that there were significant differences between the two populations of migrants. The work illuminated the question of the extent to which research findings from a particular location or setting may be relevant elsewhere, within or across countries, and linkage of particular methodologies to the research problem. A commonality was that in both countries there was a strong need to increase research and knowledge about the health of middle-aged and older migrants, since this is crucial for judging the future health care burden in culturally diverse and ageing populations, in order to better inform policies and interventions in this field.

In planning future research, there is need to decide which kinds of migrants to investigate (generations, sources, choice of which health problems to look at—mental health, injuries, NCDs, etc.—and diverse settings spanning from new arrivals to those integrated in the host population) and address common designs and methodologies. There is a scarcity of funding to support international public health research on migrants and refugees and more could be done to assist this, for example through the EU Framework Programme.

### Leveraging information technology (IT): eHealth records

Faced with the magnitude of challenges presented by the large numbers of migrants and refugees globally and the range of health problems that need to be addressed, there is a compelling case for making the best possible use of IT, both to manage health provision and as a tool for generating aggregate data and for exploring research questions.

A study reported at the M8 Alliance Expert Meeting in Rome exemplified how eHealth records could assist. In Bavaria, responsibility for management of the health of refugees eventually passes from the reception or residential centre to the general practitioner. However, there are often delays in transfer of data from the reception centres, important details may be missing and there can be critical gaps regarding contagious diseases, leading to risks of contagion, risks for health care professionals and duplication of medical examinations effort that can increase costs as well as raise risks to patients [e.g. repeating X-rays]. CompuGroup Medical, a leading company in medical information technology, worked with the Bayerische TelemedAllianz to produce electronic health records for refugees and asylum seekers. These ensure that all doctors involved in the treatment process of a refugee can access the complete and up-to-date health information of this person at anytime and anywhere. Access codes ensured privacy for patients and access for health workers. Doctors received the access code and permission from patients when required. Information was held in the system in 13 languages and there was user-friendly web access to data files when required. The system is simple and non-bureaucratic, valuable for both receiving countries and refugees, and useful in cross-border settings if the refugee moves. It has potential, therefore, to be expanded into a European project.

### Towards an agenda of solutions

During the M8 Alliance Expert Meeting in Rome, as well as in recent papers and other meetings [177, 244–246], emphasis was placed on the need to reset the agenda and turn the focus of attention regarding migrants’ and refugees’ health from an agenda of conflicts and problems into one of solutions.

As set out in the WHO-EURO Strategy and Action Plan for Refugee and Migrant Health, the scope of such an agenda should be designed to respond to the health needs associated with the migration process, namely, the need to ensure the availability, accessibility, acceptability, affordability and quality of essential services in transit and host environments, including health and social services, together with basic services such as water and sanitation, as well as addressing vulnerability to health risks, exposure to potential hazards and stress and increased susceptibility to poverty and social exclusion, abuse and violence and stigmatisation.

In terms of guiding principles, Diaz et al. [177] suggested four specific goals for a solution-based agenda: expand the scope from perceived homogeneity to social cohesion, move from description to intervention, strive for a comprehensive view of migration and implement the agenda.

Khan et al [247] proposed three important contributions that the global health community can make, with a particular focus on the refugee crisis in Europe. First, policy decisions should be based on sound evidence regarding health risks and burdens to health systems, rather than prejudice or unfounded fears. Second, for incoming refugees, the focus must be on building inclusive, cost-effective health services to promote collective health security. Third, alongside protracted conflicts, widening health and socioeconomic inequalities between high-income and lower-income countries should be acknowledged as major drivers for the global refugee crisis and fully considered in planning long-term solutions.

A combination of points from the M8 Alliance Expert Meeting and from the literature suggests that an agenda of solutions should include the following:

#### Framing

The available evidence indicates that substantial migration movements are going to remain a major and long-term facet of the globalised world in the twenty-first century, driven by a diverse array of nature- and human-related causes. By contrast, much of the current framing of migration, seen in the press, social media and policies in many countries, remains rooted in a paradigm of immobility as the norm and migration as an exceptional circumstance to be responded to as a short-term emergency.

An essential component of the transformation of the perspective to an agenda of solutions is therefore a metamorphosis that shifts the framing of migration:

- So that migration and diversity is perceived as normality, as a long-term, if not permanent, structural process that the world as a whole needs to accommodate—including the framing of ‘environmental migration’ as a necessary and positive adaptation to climate and environmental changes and extreme weather events that are being made more frequent by global warming;
- To promote understanding that, as humanitarian action to settle those displaced by disasters, environmental changes, conflict, persecution or inequity is developed, policies and practices need to be adopted, in accord with international instruments, that enable migrants and refugees to move safely without persecution or abuse; be accorded protection, care and essential health services at transit and short-term settlement points; and be given affordable access to a full range of health services to meet their physical and mental health needs where they are hosted in the longer term;
- To raise awareness that migrants and refugees, including economic migrants, can bring significant benefits to the countries that host them, including improved demographics and heightened economic activity and productivity; and
- To promote recognition that the adaptation of health (and other) services to make them inclusive and migrant-friendly not only ensures that migrants’ health problems are adequately treated, but also has positive impacts on the quality, efficiency and effectiveness of the services for all in the society.

Shifting the framing of discussion about migrants and refugees in general, and about their health needs as one of the essential elements, depends on several inter-related factors, including policy, evidence and communication, each of which is discussed below.

#### International collaboration and support

International cooperation to address the immediate health needs of migrants and refugees on the move and in temporary transit locations and those residing in longer-term facilities close to their country of origin is necessary to meet humanitarian requirements. International cooperation is also important, along with taking other measures such as ensuring opportunities for safe and decent living, education and earning livelihoods, in efforts to reduce the incentives to undertake long and potentially perilous journeys to seek refuge elsewhere. Collaboration and support, driven by political will and commitment, needs to ensure that there are plans and material and financial resources in place that can be rapidly drawn on to meet the immediate needs of sudden crises and that adequate provision is made for the ongoing needs of migrants and refugees in longer term residence facilities, including for a full range of health services.

Among the key elements that should be included in the international cooperation are:

- Development and implementation of standardised procedures for health assessment and care.
- Strongly inter-sectoral approaches that take into account the all-round wellbeing of the migrants and refugees, including attention to factors such as nutrition, clean water, sanitation, shelter, transport and education facilities, as well as to medically and culturally appropriate health services.
- Cross-border collaboration in research, education and training, as discussed below.

#### Policy

Migration is a major social, political and public health challenge, and policy makers need to develop specific and coherent policies addressing the health needs of migrants independently from their legal status. Emphasis should be placed on the approaches required to meet the diverse needs of refugees, asylum seekers and migrants at different stages, addressing the immediate and long-term health requirements, as well as public health aspects.

In achieving this, it is important that policy makers are encouraged to:

- Recognise migration as a structural phenomenon in the twenty-first century, which requires policies to prepare for both ongoing migration and for crises;
- Understand the totality of reasons for migration;
- Act in accordance with the shared responsibility of the international community expressed in the conventions, charters and resolutions to which they are signatories, including recognising rights to health and health care, and implement the New York Declaration for refugees and Migrants and, as relevant, regional instruments such as the Strategy and action plan for Refugee and Migrant Health in the WHO European Region;
- Develop and implement national policies based on principles of equity that ensure entitlement to affordable health care for all those in need in society, including migrants, refugees and asylum seekers;
- Accept the evidence for the benefits that accrue from the inclusion of migrants and refugees into mainstream societies, including providing entitlement to the full range of health services accessible to the general population and plan and implement systems to this information;
- Support work to study the health of migrants and refugees, including the systematic, routine collection of data and research to understand health issues and to develop and explore the effectiveness of prevention, treatment and rehabilitation approaches.
- Promote an inter-sectorial approach and cross-border cooperation, including in approaches to public health and action on the social determinants of health;
- Engage in communicating these positions and understandings to the public and media, disseminating and explaining them and undertaking efforts to increase public awareness about rights and responsibilities; and
- Demonstrate the political will and commitment to translating them into evidence-based policies, strategies and practices.

#### Health services and the health and wellbeing of migrants and refugees

Areas where attention and resources need to be focused in order to meet current and future challenges include:

- Develop and implement strategies to utilise the social determinants of health framework in identifying the health needs of migrants and refugees and devising and implementing solutions.
- Deal with physical and mental health issues early [247]. As noted by the World Economic Forum, poor health affects a migrant’s ability to get a job, learn the local language, interact with public institutions and do well in school—all things that are critical to integrating successfully, with refugees particularly prone to mental health issues such as anxiety and depression, following their often traumatic and violent experiences back home and in flight. Host countries should assess the mental health of newcomers alongside physical evaluations, grant humanitarian migrants access to regular health care and ensure they are able to use it.
- Provide cultural/transnational competence education and training for health care providers as preparation for ethnically and socially discordant clinical encounters and improve communication services for them in order to promote an inclusive and culturally sensitive health system;
- Ensure that culturally competent interpreters are available on-tap through communication linkages, to facilitate engagement between health workers and migrants. This is especially crucial in addressing mental health issues of migrants, including child migrants who may have been traumatised.

#### Communication

Communication is central to the engagement between the diverse actors that participate in and influence the health of migrants. Critical pathways of communication include those among and between health professionals, migrants, policy makers, researchers, the public and the media.

- The agenda for communication must not be left, by default, to the whims of particular media or political actors seeking to use migration to promote their own purposes. There needs to be systematic development of communication strategies and assets, including sound data and evidence, which are oriented to the welfare, health and wellbeing of migrants.
- The voices of migrants themselves need to be included, and, among other measures, this requires ensuring the availability of culturally competent interpreters.

#### Education and training

- Incorporate migrant and refugee health in curricula of medicine and public health degrees, courses and professional development programmes.
- Launch specific training programmes for health care professionals.
- Expand the availability of degrees and modules on migrant and refugee health, including through the use of web-based, open access materials and distance education tools such as MOOCs.
- In all materials, place emphasis on the need to develop cultural competence as well as technical skills in medical diagnosis and treatment.
- As part of the longer term aspect of the ‘agenda for solutions’, effort needs to be made on the training of trainers—giving health professionals skills to be able to train people for dealing with the particular health problems of migrants and refugees.

#### Academia: research and public engagement

Academia has unique capacities to fill the gaps in the present lack of knowledge about the health status and needs of migrants and refugees. It also has responsibility to communicate its findings, as a basis for evidence-informed policy and as a shared responsibility with the government, to ensure that the public are well informed and able to distinguish between fact, conjecture and opinion in forming their views about migration.

Recommendations for action include:

- Prioritised research agendas need to be developed at national and international levels that comprehensively address short-, medium- and long-term aspects of migrants’ and refugees’ health, across the spectrum of physical and mental health and wellbeing and within a broad social determinant of health framework. These research agendas should be participatory and, as appropriate, multi-, inter- or trans-disciplinary.
- National and international resources need to be directed to supporting this broad spectrum of research relevant to the health of migrants and refugees. The priority and value of such research (which is related to funding decisions, publication channels, academic credit and career progression) needs to be judged on its relevance and value to addressing the health needs of migrants and refugees rather than only on conventional ‘scientific excellence’ grounds.
- Research should be encouraged in the field of monitoring and assessing the impact on the health of migrants and refugees of policies in multiple sectors, including the development of specific tools and indicators.
- An essential dimension that should be built into all research in the field is a requirement to communicate the results to the public, policy makers and media in ways that will encourage attention and facilitate understanding of the conclusions and relevance.
- Further research into the evidence/media coverage/public attitudes/policy/practice nexus should be undertaken, to provide better understanding of the inter-relationships and of effective communication strategies.
- Academics must be prepared to use their voice, individually and collectively, to speak out when incorrect facts or interpretations are being offered or when policies or practices are contrary to available evidence and likely to be inimical to the health of migrants and refugees.
- National academies and professional associations, across the whole spectrum of medical and health-related disciplines, have a central role to play in promoting and facilitating all of these areas of action and of engaging themselves with researchers, funders, policy makers, the media and public to help drive forward the agenda for solutions.

The model of the Franco-German collaboration that established the Centre Virchow-Villermé Paris-Berlin, which, among other areas, has studied the inter-relationships between climate change, migration and health, could be extended with other inter-country collaborations where there is already strong engagement, e.g. between Germany and Italy.

The M8 Alliance, which has expanded its membership to 25 based in 18 countries, also has a wider collaborative network of academic institutions known for educational and research excellence, including national academies of science from 97 countries [248].

Such initiatives need to be extended, mirrored and, above all, given more resources in order to serve as a major international resource for evidence on the health issues of migrants and refugees and the effectiveness of measures implemented to meet their health needs. Key international actors, including WHO, UNHCR, IOM and ILO as well as major humanitarian NGOs, need to be drawn into participation in these initiatives. Through developing a community of practice, these initiatives can also provide a coordinated network for interaction, enabling sharing of diverse experiences and approaches and facilitating the effective use of research capacities.

## Conclusion

It is evident that migration has become a structural phenomenon of the twenty-first century, necessitating that policy makers move from a paradigm of immobility, recognise the totality of reasons for migration and examine the consequences for different sectors, with implications for planning, budgeting and implementation.

In the health field, it is crucial that the needs of migrants and refugees be met within the framework of international agreements which countries have adopted, recognising the right to health of all individuals and the centrality of this principle in the design and implementation of policies and programmes at global, regional, national and local levels.

The ‘agenda of solutions’ presented here aims to provide a comprehensive framework, which bridges humanitarian, ethical and rights-based imperatives to provide a framework for action to tackle this crucial area. It stresses the need for:

- Framing the issue of migration as a normal and constructive response to the realities of local circumstances in many places in the world—i.e. as an adaptation strategy;
- Intensified international collaboration and support to address both immediate and long-term health needs of migrants;
- Policies that are based on principles of the right to health, equity and social justice and that recognise the benefits to all of ensuring the inclusion of migrants and refugees into mainstream societies, including providing entitlement to the full range of health services accessible to the general population;
- Utilising the social determinants of health framework in identifying the health needs of migrants and refugees and devising and implementing solutions;
- Dealing with physical and mental health issues early;
- Developing cultural/transnational competence in health professionals, their institutions and agencies working with migrants and refugees;
- Ensuring the availability of culturally competent interpreters in engagements between migrants and refugees and health workers;
- Developing a communications approach oriented to the welfare, health and wellbeing of migrants, including through the inclusion of the voices of migrants themselves;
- Expanding the education and training of health professionals working with migrants and refugees and of migrants and refugees who are health professionals, to enable them to work in their fields of expertise for the benefit of themselves, the migrant and refugee community and their host countries;
- Supporting prioritised research agendas that comprehensively address migrants’ and refugees’ health, across the spectrum of physical and mental health and wellbeing and within a broad social determinant of health framework;
- Encouraging academics to use their voice to speak out regarding facts, interpretations, policies and practices to promote the health of migrants and refugees;
- Encouraging national academies and professional associations to play an active role at national, regional and global levels.
- Promoting a twin-track approach that addresses the root causes of displacement while dealing with the chronic problem of managing the safety, welfare and health of those displaced.

In the closing session of the M8 Alliance Expert Meeting, Ambassador Hinrich Thölken of Germany commented that ‘we cannot accept a bleak future: we can do better…. We must remain optimistic and keep a positive spirit – as a tool, even a strategy.’

## Abbreviations

CBR: Community-based rehabilitation
EC: European Commission
EU: European Union
HIV: Human immunodeficiency virus
ILO: International Labour Organization
IOM: International Organization for Migration
IT: Information technology
MOOCs: Massive Open Online Courses
NCDs: Non-communicable diseases
NGO: Non-governmental organisation
OECD: Organisation for Economic Cooperation and Development
SIMM: Italian Society of Migration Medicine
TB: Tuberculosis
UHPU: University Hospital Policlinico Umberto I
UK: United Kingdom
UN: United Nations
UNHCR: United Nations High Commissioner for Refugees
USA: United States of America
WHO: World Health Assembly
WHO: World Health Organization
WHO-EURO: World Health Organization European Region
